# A systematic review of influences on engagement with remote health interventions targeting weight management for individuals living with excess weight

**DOI:** 10.1038/s41366-025-01811-8

**Published:** 2025-06-07

**Authors:** Jamie M. Whitehall, Erica J. Cook, Jitka Vseteckova, Kerry Jones, Yannis Pappas, Louisa Donald, Angel M. Chater

**Affiliations:** 1 Research Centre for Health Service Organisation and Delivery, Institute for Health Research, University of Bedfordshire, Vicarage Street, Luton, LU1 3JU United Kingdom; 2 Centre for Health Wellbeing and Behaviour Change, Institute for Sport and Physical Activity Research, University of Bedfordshire, Polhill Avenue, Bedford, MK41 9EA United Kingdom; 3 School of Psychology, University of Bedfordshire, Vicarage Street, Luton, LU1 3JU United Kingdom; 4 Faculty of Wellbeing Education & Language Studies, The Open University, Milton Keynes, United Kingdom; 5 Centre for Behaviour Change, University College London, 1-19 Torrington Place, London, WC1E 7HB United Kingdom

**Keywords:** Weight management, Public health

## Abstract

**Background:**

Obesity rates are continually rising and remote weight management interventions appear to demonstrate feasible prospects. Previous reviews have investigated influential factors to engagement of such interventions in community settings; however, limited research has examined adults’ engagement in remote weight management programmes.

**Aim:**

To systematically review the influences on the engagement of adults living with excess weight in synchronous (real time), remote health interventions for weight management.

**Methods:**

A systematic review of 12 databases was conducted from inception to October 2023. Studies were included if they delivered a synchronous, remote weight management intervention with participants that were ≥18 years old with a body mass index ≥27.5 kg/m^2^. A narrative synthesis with inductive thematic analysis was conducted to iteratively extrapolate barriers and facilitators to engagement. This set of themed influences were then deductively mapped to the COM-B model of behaviour change and the Theoretical Domains Framework (TDF).

**Results:**

From 36,359 studies screened, 39 studies met the inclusion criteria. A total of 57 themed influences were iteratively coded and mapped to the COM-B model: physical capability (*n* = 2); psychological capability (*n* = 10), reflective motivation (*n* = 17); automatic motivation (*n* = 10); physical opportunity (*n* = 7); and social opportunity (*n* = 11) with the assistance of the TDF to guide the coding. Barriers to engagement (*n* = 18) included concerns surrounding privacy, time burden to engage, embarrassment/anxiety surrounding self-disclosure, technical issues, access to technology, and access to the internet. Facilitators to engagement (*n* = 39) included digital competency, familiarity with technology, self-monitoring, tailored feedback, convenience, accountability, regular check-ins, support from a professional, social support, peer support, ease of use and simplicity.

**Conclusion:**

There are a number of things to consider in relation to capability, opportunity and motivation when designing remote weight management interventions. This review provides evidence to specific barriers and facilitators that if addressed could optimise future efforts.

## Introduction

Obesity represents one of the most significant public health challenges of the twenty-first century [1]. In 2021, an estimated 64% of adults in the United Kingdom were living with excess weight, 26% of whom were living with obesity [2]. Excess weight can have a significant adverse effect on an individual’s health status and quality of life, associated with increased risk towards a plethora of long-term conditions and co-morbidities such as type 2 diabetes, cardiovascular disease, and some cancers [3–5]. Excess weight and obesity are reported as a leading cause of preventable mortality, placing a considerable strain on health systems and substantial associated economic costs [6–8].

The benefits of weight loss for individuals living with obesity have been extensively reported, ranging from individual health to global economic factors [9]. Ensuring effective strategies are established and implemented to support individuals to facilitate weight management is crucial. Weight management interventions incorporating specific behaviour change techniques have been associated with significantly greater weight loss than interventions where such techniques are absent; for example when coded to the refined behaviour change techniques taxonomy CALO-RE, in a univariate model, each additional technique used in the ‘comparison of behaviour’ domain (03: provide information about others’ approval; 04: provide normative information about others’ behaviour; 22: model or demonstrate the behaviour; and 28: facilitate social comparison) was associated with an additional −1.5 kg weight loss at 12 months [10]. Also, technique 22 ‘model or demonstrate the behaviour’ was associated with −2.7 kg greater weight loss at 12 months when controlling for the other three techniques in this domain [10]. Clinically significant weight loss (−5% of body weight) has beneficial impacts, however, weight regain is a common clinical concern, particularly when sessions are missed or the support ceases [11]. Consequently, engagement in, adherence to and subsequent behaviour change long-term is important for weight-loss maintenance [12]. However, evidence demonstrates a large diversity in approaches and content between weight management interventions, which can negatively affect engagement and lead to variations in the effectiveness [13, 14].

Remote digital health behaviour interventions for weight management have become increasingly popular, driven by increased access to and adoption of technology over recent years [15, 16]. Digital approaches to weight management have been shown to have comparable effectiveness to face-to-face delivery regarding weight reduction outcomes [17, 18]. The COVID-19 pandemic has provided a unique opportunity to deliver more cost-effective, convenient and scalable digital solutions [19, 20], particularly among underserved populations who have been disproportionately affected by obesity [21] and COVID-19 [22]. There are, however, increasing concerns that the movement towards digital solutions to weight loss may lead to digital exclusion and exacerbate pre-existing health inequalities [23], with ethnically diverse, older, and economically disadvantaged populations shown to be less likely to engage with remote interventions [24–26].

The engagement of digital behaviour change interventions has been conceptualised as both a behaviour (e.g., attendance dose, attendance frequency, attendance duration, depth of usage) and subjective experience, which draws on users’ cognitive and emotional states when interacting with the intervention (e.g., attention, interest and affect) [27]. The barriers and facilitators to engaging with remote weight management interventions remain unclear [23, 28, 29] and this is the focus of this review.

## Methods

This systematic review used the Preferred Reporting Items for Systematic Review and Meta-Analysis (PRISMA) checklist guidelines [30, 31]. The protocol is registered in PROSPERO [CRD42021253439] [32]. This review was guided by methods highlighted by the Cochrane Handbook for systematic reviews [33]. Ethical approval was granted by The University of Bedfordshire Institute for Sport and Physical Activity Research Ethics Committee (REF: 2021ISPAR009).

### Inclusion criteria

The Population, Intervention, Comparison, Outcomes, and Study design (PICOS) framework was used to define the eligibility criteria [34]. The following criteria were selected for inclusion.

#### Population

Studies including Adults (>18 years old) living with excess weight using the adjusted BMI obesity category thresholds to be inclusive of all ethnic groups (BMI ≥ 27.5 kg/m^2^) [35] and/or providers of care to this population following the NICE guidelines.

#### Intervention

Studies that included a synchronous (real-time), interactive, remotely delivered weight management intervention.

#### Comparison

Studies with or without a comparison group.

#### Outcome(s)

Studies that reported on the barriers and facilitators to engage with a remotely delivered weight management intervention.

#### Study design

Mixed-methods, quantitative and qualitative study designs where barriers and facilitators to engaging in a synchronous, interactive, remotely delivered weight management intervention were measured/discussed.

### Exclusion criteria

The following criteria were set for exclusion using the PICOS framework.

#### Population

Studies with participants <18 years of age or with a BMI < 27.5 kg/m^2^ were excluded.

#### Intervention

Studies that were not a health intervention with a weight management focus, alongside those that were solely autonomous or delivered only face-to-face, were excluded. Studies that reported predicting factors (such as demographic predictors) without specific barriers or facilitators were also excluded.

#### Comparison

No exclusion specification was set for this criteria.

#### Outcome(s)

Studies not specifically reporting barriers or facilitators to engagement in a remote weight management intervention

#### Study design

Studies not published in the English Language and systematic reviews, conference papers, protocols, case studies, opinion and editorial letters, and unpublished works were excluded.

### Search strategy

A systematic literature search strategy of 12 electronic databases including grey literature (MEDLINE, PubMed, CINAHL, PsycINFO, ProQuest, Scopus, Web of Science, SPORTDiscus, ISRCTN, NHS Evidence, ClinicalTrials.gov, and CENTRAL) was formulated, agreed upon between all authors and conducted from inception up to and including October 2023. The search strategy was co-developed with the research team and an experienced subject specialist librarian was derived from key terms, their synonyms, and MeSH terms, which were combined using Boolean operators (AND, OR) for terms relating to the population, intervention, and outcomes (see Box 1). Results were then filtered to include only the English language. Reference lists of any relevant/included studies were also hand-searched and screened for inclusion.

Box 1 Search strategy **P:** (Adult) [MESH]

AND

(Obesity OR obese OR overweight OR weight OR BMI)

**I:** AND (Remote OR online OR internet OR app OR digital OR telehealth OR mobile OR phone OR telemedicine OR mHealth or e-health OR web-based OR social networking OR Facebook OR messenger OR WhatsApp OR interactive OR synchronous)

AND

(Weight OR intervention OR behaviour change OR lifestyle)

**C:** *No specification in search query*

**O:** AND (Barriers OR facilitators OR engagement OR uptake OR dropout OR acceptability OR feasibility OR enablers OR adherence)

AND

(Weight loss OR dietary change OR physical activity OR well-being OR lifestyle)

### Study selection and data extraction

**Fig. 1:**
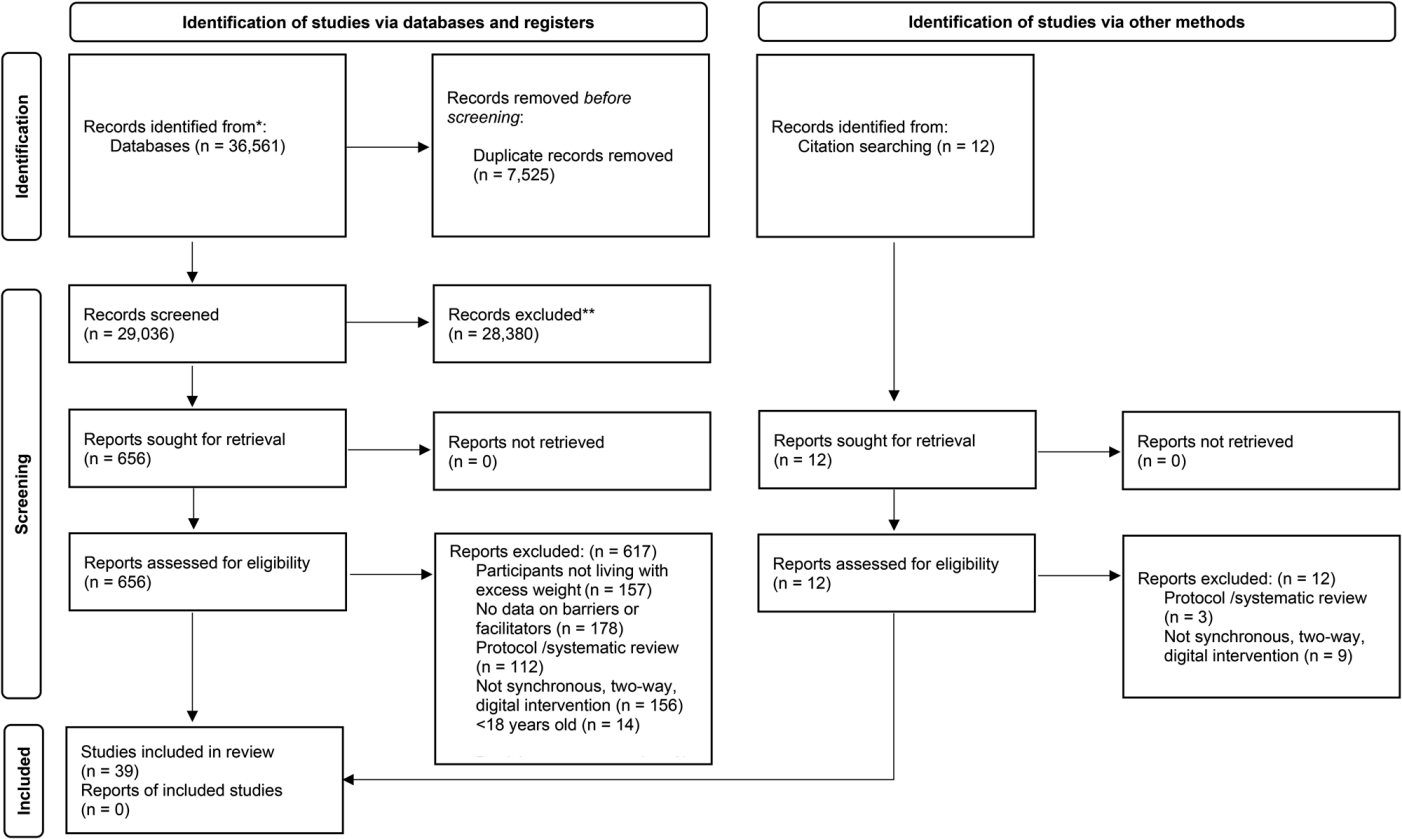
PRISMA flow diagram for study inclusion. This figure illustrates the different phases of the systematic review, specifically the number of records identified, screened, assessed for eligibility, and included in the final analysis.

Retrieved articles were exported into Rayyan software [36] with duplicates being identified and removed. Titles and abstracts were then evenly distributed between all authors and were double-screened for eligibility using the inclusion and exclusion criteria, with any discrepancies discussed and resolved by three authors (JMW, JV, and AMC) to reach a consensus. The full-text screening was conducted similarly, with discrepancies resolved by three authors (JMW, JV, and AMC). A PRISMA flow diagram was used to report the screening process [31] (see Fig. 1).

A data extraction template was developed using Microsoft Excel, which included general information (authors, publication year), country, study characteristics (design, aims), participant characteristics (sample size, age, gender/sex, ethnicity, socio-economic status, education), intervention features (descriptions of the intervention including duration, setting, provider, delivery format and procedure using TIDieR guidelines [37]), outcomes (including; consent/attrition/drop out, barriers, facilitators) which was completed by one researcher (EC) and checked by JMW.

### Theoretical underpinnings

The use of behaviour change theory has been strongly recommended when designing, delivering and evaluating health interventions, particularly when assessing barriers and facilitators. The COM-B [38] (Capability (physical and psychological), Opportunity (physical and social), Motivation (reflective and automatic)—Behaviour) model assesses individuals, capability, opportunity and motivation towards a target behaviour which occurs through an interactive system at the hub of the Behaviour Change Wheel (BCW [38]). Using the Theoretical Domains Framework (TDF), the COM-B constructs can be understood at a more granular level. Capability consists of influences such as functional ability, knowledge, skills, memory, attention, decision processes and behavioural regulation. Opportunity consists of influences such as environmental context and resources, physical time, finances, accessibility, social influences and support. Motivation consists of factors such as beliefs about capabilities, beliefs about consequences, optimism, social/professional role and identity, intentions, goals reinforced habits, and emotions.

### Data synthesis and analysis

The main analysis focused on conceptualising the barriers and facilitators to engagement, with deeper understanding though a COM-B/TDF ‘behavioural analysis’. Narrative analysis was conducted through the employment of inductive thematic synthesis to iteratively extrapolate the description of barriers and facilitators to engagement [39]. The coded data was then collated and investigated for overarching themes, which were then deductively mapped to the COM-B model of behaviour change [38]. This was conducted by one author (EC) and then independently reviewed by two other authors (AMC, JMW), and corrected for inconsistencies. All reviewers are experienced in behaviour change research with expertise using the Behaviour Change Wheel [38].

### Quality assessment

The Mixed Methods Appraisal Tool version 2018 (MMAT-v2018) [40] was utilised for its versatility of application to assess a multitude of study designs, including qualitative, randomised controlled, nonrandomized, quantitative descriptive, and mixed methods, being the only tool to include specific criteria for appraising the quality of integrated qualitative and quantitative, mixed-methods studies. This was independently applied by two authors (YP, JV), assessing study quality and risk of bias by answering “yes”, “no”, or “can’t tell” to two screening questions and five key questions based on the study’s methodological approach. Once completed, ratings were individually assessed by a third author (JMW) to resolve discrepancies and reach a consensus. All studies satisfied the two initial screening questions of the MMAT-V 2018 and were generally of high quality, with all studies satisfying more than 50% of the appropriate appraisal criteria. Very few studies had criteria deemed as not satisfied, and where it occurred, it mostly related to an underlying reason, such as not being possible due to being a feasibility or pilot study. An overall numerical MMAT-V 2018 score for each study was not calculated as the score does not highlight the problematic areas of the study in terms of quality [41]; instead, a more in-depth explanation of each specific criterion has been reported [40] (see Table 1).

**Table 1:** Study quality appraisals.

**Study: Abbott et al.** [42]					
**Category of study designs**	**Methodological quality criteria**			**Responses**	
		Yes	No	Can’t tell	Comments
Screening questions (for all types)	S1. Are there clear research questions?	x			
	S2. Do the collected data allow to address the research questions?	x			
	*Further appraisal may not be feasible or appropriate when the answer is ‘No’ or ‘Can’t tell’ to one or both screening questions*.				
3. Quantitative nonrandomized	3.1. Are the participants representative of the target population?	x			
	3.2. Are measurements appropriate regarding both the outcome and intervention (or exposure)?	x			
	3.3. Are there complete outcome data?	x			
	3.4. Are the confounders accounted for in the design and analysis?	x			
	3.5. During the study period, is the intervention administered (or exposure occurred) as intended?	x			
**Study: Abshire et al**. [43]					
**Category of study designs**	**Methodological quality criteria**			**Responses**	
		Yes	No	Can’t tell	Comments
Screening questions (for all types)	S1. Are there clear research questions?	x			
	S2. Do the collected data allow to address the research questions?	x			
	*Further appraisal may not be feasible or appropriate when the answer is ‘No’ or ‘Can’t tell’ to one or both screening questions*.				
4. Quantitative descriptive	4.1. Is the sampling strategy relevant to address the research question?	x			
	4.2. Is the sample representative of the target population?	x			
	4.3. Are the measurements appropriate?	x			
	4.4. Is the risk of nonresponse bias low?			x	
	4.5. Is the statistical analysis appropriate to answer the research question?	x			
**Study: Allen et al**. [44]					
**Category of study designs**	**Methodological quality criteria**			**Responses**	
		Yes	No	Can’t tell	Comments
Screening questions (for all types)	S1. Are there clear research questions?	x			
	S2. Do the collected data allow to address the research questions?	x			
	*Further appraisal may not be feasible or appropriate when the answer is ‘No’ or ‘Can’t tell’ to one or both screening questions*.				
2. Quantitative randomised controlled trials	2.1. Is randomisation appropriately performed?			x	
	2.2. Are the groups comparable at baseline?	x			
	2.3. Are there complete outcome data?	x			Participants with missing data were not entered into analysis
	2.4. Are outcome assessors blinded to the intervention provided?			x	may not be an issue as this was feasibility study
	2.5 Did the participants adhere to the assigned intervention?	X			
**Study: Arigo et al**. [45]					
**Category of study designs**	**Methodological quality criteria**			**Responses**	
		Yes	No	Can’t tell	Comments
Screening questions (for all types)	S1. Are there clear research questions?	x			
	S2. Do the collected data allow to address the research questions?	x			
	*Further appraisal may not be feasible or appropriate when the answer is ‘No’ or ‘Can’t tell’ to one or both screening questions*.				
4. Quantitative descriptive	4.1. Is the sampling strategy relevant to address the research question?	x			
	4.2. Is the sample representative of the target population?	X			
	4.3. Are the measurements appropriate?	x			Although small size
	4.4. Is the risk of nonresponse bias low?	x			
	4.5. Is the statistical analysis appropriate to answer the research question?	x			

**Study: Asbjornsen et al.** [46]					
**Category of study designs**	**Methodological quality criteria**			**Responses**	
		Yes	No	Can’t tell	Comments
Screening questions (for all types)	S1. Are there clear research questions?	x			
	S2. Do the collected data allow to address the research questions?	x			
	*Further appraisal may not be feasible or appropriate when the answer is ‘No’ or ‘Can’t tell’ to one or both screening questions*.				
1. Qualitative	1.1. Is the qualitative approach appropriate to answer the research question?	x			
	1.2. Are the qualitative data collection methods adequate to address the research question?	x			
	1.3. Are the findings adequately derived from the data?	x			
	1.4. Is the interpretation of results sufficiently substantiated by data?	x			
	1.5. Is there coherence between qualitative data sources, collection, analysis and interpretation?	x			
**Study: Batsis et al**. [47]					
**Category of study designs**	**Methodological quality criteria**			**Responses**	
		Yes	No	Can’t tell	Comments
Screening questions (for all types)	S1. Are there clear research questions?	x			
	S2. Do the collected data allow to address the research questions?	x			
	*Further appraisal may not be feasible or appropriate when the answer is ‘No’ or ‘Can’t tell’ to one or both screening questions*.				
4. Quantitative descriptive	4.1. Is the sampling strategy relevant to address the research question?	x			
	4.2. Is the sample representative of the target population?	x			
	4.3. Are the measurements appropriate?	x			
	4.4. Is the risk of nonresponse bias low?	x			Feasibility study—deemed low risk
	4.5. Is the statistical analysis appropriate to answer the research question?	x			
**Study: Batsis et al**. [48]					
**Category of study designs**	**Methodological quality criteria**			**Responses**	
		Yes	No	Can’t tell	Comments
Screening questions (for all types)	S1. Are there clear research questions?	x			
	S2. Do the collected data allow to address the research questions?	x			
	*Further appraisal may not be feasible or appropriate when the answer is ‘No’ or ‘Can’t tell’ to one or both screening questions*.				
1. Qualitative	1.1. Is the qualitative approach appropriate to answer the research question?	x			
	1.2. Are the qualitative data collection methods adequate to address the research question?	x			
	1.3. Are the findings adequately derived from the data?	x			
	1.4. Is the interpretation of results sufficiently substantiated by data?	x			
	1.5. Is there coherence between qualitative data sources, collection, analysis and interpretation?	x			
**Study: Batsis et al**. [49]					
**Category of study designs**	**Methodological quality criteria**			**Responses**	
		Yes	No	Can’t tell	Comments
Screening questions (for all types)	S1. Are there clear research questions?	x			
	S2. Do the collected data allow to address the research questions?	x			
	*Further appraisal may not be feasible or appropriate when the answer is ‘No’ or ‘Can’t tell’ to one or both screening questions*.				
1. Qualitative	1.1. Is the qualitative approach appropriate to answer the research question?	x			
	1.2. Are the qualitative data collection methods adequate to address the research question?	x			
	1.3. Are the findings adequately derived from the data?	x			
	1.4. Is the interpretation of results sufficiently substantiated by data?	x			
	1.5. Is there coherence between qualitative data sources, collection, analysis and interpretation?	x			
**Study: Bennett et al**. [50]					
**Category of study designs**	**Methodological quality criteria**			**Responses**	
		Yes	No	Can’t tell	Comments
Screening questions (for all types)	S1. Are there clear research questions?	x			
	S2. Do the collected data allow to address the research questions?	x			
	*Further appraisal may not be feasible or appropriate when the answer is ‘No’ or ‘Can’t tell’ to one or both screening questions*.				
2. Quantitative randomised controlled trials	2.1. Is randomisation appropriately performed?	x			
	2.2. Are the groups comparable at baseline?	x			
	2.3. Are there complete outcome data?	x			
	2.4. Are outcome assessors blinded to the intervention provided?		X		Randomisation method which precluded groups being blinded
	2.5 Did the participants adhere to the assigned intervention?	X			Missing visits were treated as missing at random and addressed using intent-to-treat principles and maximum likelihood methods.
**Study: Cliffe et al**. [51]					
**Category of study designs**	**Methodological quality criteria**			**Responses**	
		Yes	No	Can’t tell	Comments
Screening questions (for all types)	S1. Are there clear research questions?	x			
	S2. Do the collected data allow to address the research questions?	x			
	*Further appraisal may not be feasible or appropriate when the answer is ‘No’ or ‘Can’t tell’ to one or both screening questions*.				
1. Qualitative	1.1. Is the qualitative approach appropriate to answer the research question?	x			
	1.2. Are the qualitative data collection methods adequate to address the research question?	x			
	1.3. Are the findings adequately derived from the data?	x			
	1.4. Is the interpretation of results sufficiently substantiated by data?	x			
	1.5. Is there coherence between qualitative data sources, collection, analysis and interpretation?	x			
**Study: Connelly et al**. [52]					
**Category of study designs**	**Methodological quality criteria**			**Responses**	
		Yes	No	Can’t tell	Comments
Screening questions (for all types)	S1. Are there clear research questions?	x			
	S2. Do the collected data allow to address the research questions?	x			
	*Further appraisal may not be feasible or appropriate when the answer is ‘No’ or ‘Can’t tell’ to one or both screening questions*.				
5. Mixed methods	5.1. Is there an adequate rationale for using a mixed methods design to address the research question?	x			
	5.2. Are the different components of the study effectively integrated to answer the research question?	x			
	5.3. Are the outputs of the integration of qualitative and quantitative components adequately interpreted?	x			
	5.4. Are divergences and inconsistencies between quantitative and qualitative results adequately addressed?	x			
	5.5. Do the different components of the study adhere to the quality criteria of each tradition of the methods involved?	x			
**Study: Cook et al**. [53]					
**Category of study designs**	**Methodological quality criteria**			**Responses**	
		Yes	No	Can’t tell	Comments
Screening questions (for all types)	S1. Are there clear research questions?	x			
	S2. Do the collected data allow to address the research questions?	x			
	*Further appraisal may not be feasible or appropriate when the answer is ‘No’ or ‘Can’t tell’ to one or both screening questions*.				
1. Qualitative	1.1. Is the qualitative approach appropriate to answer the research question?	x			
	1.2. Are the qualitative data collection methods adequate to address the research question?	x			
	1.3. Are the findings adequately derived from the data?	x			
	1.4. Is the interpretation of results sufficiently substantiated by data?	x			
	1.5. Is there coherence between qualitative data sources, collection, analysis and interpretation?	x			
**Study: Damschroder et al**. [54]					
**Category of study designs**	**Methodological quality criteria**			**Responses**	
		Yes	No	Can’t tell	Comments
Screening questions (for all types)	S1. Are there clear research questions?	x			
	S2. Do the collected data allow to address the research questions?	x			
	*Further appraisal may not be feasible or appropriate when the answer is ‘No’ or ‘Can’t tell’ to one or both screening questions*.				
4. Quantitative descriptive	4.1. Is the sampling strategy relevant to address the research question?	x			
	4.2. Is the sample representative of the target population?	x			
	4.3. Are the measurements appropriate?	x			
	4.4. Is the risk of nonresponse bias low?	x			
	4.5. Is the statistical analysis appropriate to answer the research question?	x			
**Study: Das et al**. [55]					
**Category of study designs**	**Methodological quality criteria**			**Responses**	
		Yes	No	Can’t tell	Comments
Screening questions (for all types)	S1. Are there clear research questions?	x			
	S2. Do the collected data allow to address the research questions?	x			
	*Further appraisal may not be feasible or appropriate when the answer is ‘No’ or ‘Can’t tell’ to one or both screening questions*.				
1. Qualitative	1.1. Is the qualitative approach appropriate to answer the research question?	x			
	1.2. Are the qualitative data collection methods adequate to address the research question?	x			
	1.3. Are the findings adequately derived from the data?	x			
	1.4. Is the interpretation of results sufficiently substantiated by data?	x			
	1.5. Is there coherence between qualitative data sources, collection, analysis and interpretation?	x			
**Study: Das et al**. [56]					
**Category of study designs**	**Methodological quality criteria**			**Responses**	
		Yes	No	Can’t tell	Comments
Screening questions (for all types)	S1. Are there clear research questions?	x			
	S2. Do the collected data allow to address the research questions?	x			
	*Further appraisal may not be feasible or appropriate when the answer is ‘No’ or ‘Can’t tell’ to one or both screening questions*.				
4. Quantitative descriptive	4.1. Is the sampling strategy relevant to address the research question?	x			
	4.2. Is the sample representative of the target population?	x			
	4.3. Are the measurements appropriate?	x			
	4.4. Is the risk of nonresponse bias low?	x			
	4.5. Is the statistical analysis appropriate to answer the research question?	x			
**Study: Donnelly et al**. [57]					
**Category of study designs**	**Methodological quality criteria**			**Responses**	
		Yes	No	Can’t tell	Comments
Screening questions (for all types)	S1. Are there clear research questions?	x			
	S2. Do the collected data allow to address the research questions?	x			
	*Further appraisal may not be feasible or appropriate when the answer is ‘No’ or ‘Can’t tell’ to one or both screening questions*.				
2. Quantitative randomised controlled trials	2.1. Is randomisation appropriately performed?			x	
	2.2. Are the groups comparable at baseline?	x			
	2.3. Are there complete outcome data?	x			
	2.4. Are outcome assessors blinded to the intervention provided?			x	
	2.5 Did the participants adhere to the assigned intervention?	x			
**Study: Dutton et al**. [58]					
**Category of study designs**	**Methodological quality criteria**			**Responses**	
		Yes	No	Can’t tell	Comments
Screening questions (for all types)	S1. Are there clear research questions?	x			
	S2. Do the collected data allow to address the research questions?	x			
	*Further appraisal may not be feasible or appropriate when the answer is ‘No’ or ‘Can’t tell’ to one or both screening questions*.				
4. Quantitative descriptive	4.1. Is the sampling strategy relevant to address the research question?	x			
	4.2. Is the sample representative of the target population?	x			
	4.3. Are the measurements appropriate?	x			
	4.4. Is the risk of nonresponse bias low?	x			
	4.5. Is the statistical analysis appropriate to answer the research question?	x			
**Study: Gibson et al**. [59]					
**Category of study designs**	**Methodological quality criteria**			**Responses**	
		Yes	No	Can’t tell	Comments
Screening questions (for all types)	S1. Are there clear research questions?	x			
	S2. Do the collected data allow to address the research questions?	x			
	*Further appraisal may not be feasible or appropriate when the answer is ‘No’ or ‘Can’t tell’ to one or both screening questions*.				
2. Quantitative randomised controlled trials	2.1. Is randomisation appropriately performed?	x			
	2.2. Are the groups comparable at baseline?	x			
	2.3. Are there complete outcome data?	x			
	2.4. Are outcome assessors blinded to the intervention provided?	x			
	2.5 Did the participants adhere to the assigned intervention?	x			
**Study: Goodrich et al**. [60]					
**Category of study designs**	**Methodological quality criteria**			**Responses**	
		Yes	No	Can’t tell	Comments
Screening questions (for all types)	S1. Are there clear research questions?	x			
	S2. Do the collected data allow to address the research questions?	x			
	*Further appraisal may not be feasible or appropriate when the answer is ‘No’ or ‘Can’t tell’ to one or both screening questions*.				
5. Mixed methods	5.1. Is there an adequate rationale for using a mixed methods design to address the research question?	x			
	5.2. Are the different components of the study effectively integrated to answer the research question?	x			
	5.3. Are the outputs of the integration of qualitative and quantitative components adequately interpreted?	x			
	5.4. Are divergences and inconsistencies between quantitative and qualitative results adequately addressed?	x			
	5.5. Do the different components of the study adhere to the quality criteria of each tradition of the methods involved?	x			
**Study: Haggerty et al**. [61]					
**Category of study designs**	**Methodological quality criteria**			**Responses**	
		Yes	No	Can’t tell	Comments
Screening questions (for all types)	S1. Are there clear research questions?	x			
	S2. Do the collected data allow to address the research questions?	x			
	*Further appraisal may not be feasible or appropriate when the answer is ‘No’ or ‘Can’t tell’ to one or both screening questions*.				
2. Quantitative randomised controlled trials	2.1. Is randomisation appropriately performed?			x	
	2.2. Are the groups comparable at baseline?	x			
	2.3. Are there complete outcome data?	x			
	2.4. Are outcome assessors blinded to the intervention provided?			x	
	2.5 Did the participants adhere to the assigned intervention?	x			
**Study: Harvey-Berino et al**. [62]					
**Category of study designs**	**Methodological quality criteria**			**Responses**	
		Yes	No	Can’t tell	Comments
Screening questions (for all types)	S1. Are there clear research questions?	x			
	S2. Do the collected data allow to address the research questions?	x			
	*Further appraisal may not be feasible or appropriate when the answer is ‘No’ or ‘Can’t tell’ to one or both screening questions*.				
4. Quantitative descriptive	4.1. Is the sampling strategy relevant to address the research question?	x			
	4.2. Is the sample representative of the target population?	X			Randomised but not an RCT
	4.3. Are the measurements appropriate?	x			
	4.4. Is the risk of nonresponse bias low?	X			Reasonable attrition
	4.5. Is the statistical analysis appropriate to answer the research question?	x			
**Study: Harvey-Berino et al**. [63]					
**Category of study designs**	**Methodological quality criteria**			**Responses**	
		Yes	No	Can’t tell	Comments
Screening questions (for all types)	S1. Are there clear research questions?	x			
	S2. Do the collected data allow to address the research questions?	x			
	*Further appraisal may not be feasible or appropriate when the answer is ‘No’ or ‘Can’t tell’ to one or both screening questions*.				
4. Quantitative descriptive	4.1. Is the sampling strategy relevant to address the research question?	x			
	4.2. Is the sample representative of the target population?	X			Randomised but not an RCT
	4.3. Are the measurements appropriate?	x			
	4.4. Is the risk of nonresponse bias low?	X			Reasonable attrition
	4.5. Is the statistical analysis appropriate to answer the research question?	x			
**Study: Harvey-Berino et al**. [64]					
**Category of study designs**	**Methodological quality criteria**			**Responses**	
		Yes	No	Can’t tell	Comments
Screening questions (for all types)	S1. Are there clear research questions?	x			
	S2. Do the collected data allow to address the research questions?	x			
	*Further appraisal may not be feasible or appropriate when the answer is ‘No’ or ‘Can’t tell’ to one or both screening questions*.				
4. Quantitative descriptive	4.1. Is the sampling strategy relevant to address the research question?	x			
	4.2. Is the sample representative of the target population?	X			Randomised but not an RCT
	4.3. Are the measurements appropriate?	x			
	4.4. Is the risk of nonresponse bias low?	X			Reasonable attrition
	4.5. Is the statistical analysis appropriate to answer the research question?	x			
**Study: Haste et al**. [65]					
**Category of study designs**	**Methodological quality criteria**			**Responses**	
		Yes	No	Can’t tell	Comments
Screening questions (for all types)	S1. Are there clear research questions?	x			
	S2. Do the collected data allow to address the research questions?	x			
	*Further appraisal may not be feasible or appropriate when the answer is ‘No’ or ‘Can’t tell’ to one or both screening questions*.				
2. Quantitative randomised controlled trials	2.1. Is randomisation appropriately performed?	X			Listed as pilot RCT
	2.2. Are the groups comparable at baseline?	x			
	2.3. Are there complete outcome data?	X			Only reasonable dropout noted
	2.4. Are outcome assessors blinded to the intervention provided?		X		not possible due to this being a pilot
	2.5 Did the participants adhere to the assigned intervention?	X			Only reasonable dropout noted
**Study: Hu et al**. [66]					
**Category of study designs**	**Methodological quality criteria**			**Responses**	
		Yes	No	Can’t tell	Comments
Screening questions (for all types)	S1. Are there clear research questions?	x			
	S2. Do the collected data allow to address the research questions?	x			
	*Further appraisal may not be feasible or appropriate when the answer is ‘No’ or ‘Can’t tell’ to one or both screening questions*.				
2. Quantitative randomised controlled trials	2.1. Is randomisation appropriately performed?	x			
	2.2. Are the groups comparable at baseline?	x			
	2.3. Are there complete outcome data?	X			Reasonably
	2.4. Are outcome assessors blinded to the intervention provided?			X	Limited information, but from what is reported, appears low concern
	2.5 Did the participants adhere to the assigned intervention?	X			Reasonably, and raised in the discussion
**Study: Job et al**. [67]					
**Category of study designs**	**Methodological quality criteria**			**Responses**	
		Yes	No	Can’t tell	Comments
Screening questions (for all types)	S1. Are there clear research questions?	x			
	S2. Do the collected data allow to address the research questions?	x			
	*Further appraisal may not be feasible or appropriate when the answer is ‘No’ or ‘Can’t tell’ to one or both screening questions*.				
1. Qualitative	1.1. Is the qualitative approach appropriate to answer the research question?	x			
	1.2. Are the qualitative data collection methods adequate to address the research question?	x			
	1.3. Are the findings adequately derived from the data?	x			
	1.4. Is the interpretation of results sufficiently substantiated by data?	x			
	1.5. Is there coherence between qualitative data sources, collection, analysis and interpretation?	x			
**Study: Kolodziejczyk et al**. [68]					
**Category of study designs**	**Methodological quality criteria**			**Responses**	
		Yes	No	Can’t tell	Comments
Screening questions (for all types)	S1. Are there clear research questions?	x			
	S2. Do the collected data allow to address the research questions?	x			
	*Further appraisal may not be feasible or appropriate when the answer is ‘No’ or ‘Can’t tell’ to one or both screening questions*.				
4. Quantitative descriptive	4.1. Is the sampling strategy relevant to address the research question?	X			Sample small—pilot feasibility study
	4.2. Is the sample representative of the target population?	x			
	4.3. Are the measurements appropriate?	x			
	4.4. Is the risk of nonresponse bias low?	x			
	4.5. Is the statistical analysis appropriate to answer the research question?	x			
**Study: Latinen et al**. [69]					
**Category of study designs**	**Methodological quality criteria**			**Responses**	
		Yes	No	Can’t tell	Comments
Screening questions (for all types)	S1. Are there clear research questions?	x			
	S2. Do the collected data allow to address the research questions?	x			
	*Further appraisal may not be feasible or appropriate when the answer is ‘No’ or ‘Can’t tell’ to one or both screening questions*.				
1. Qualitative	1.1. Is the qualitative approach appropriate to answer the research question?	x			
	1.2. Are the qualitative data collection methods adequate to address the research question?	x			
	1.3. Are the findings adequately derived from the data?	x			
	1.4. Is the interpretation of results sufficiently substantiated by data?	x			
	1.5. Is there coherence between qualitative data sources, collection, analysis and interpretation?	x			
**Study: Lewis et al**. [70]					
**Category of study designs**	**Methodological quality criteria**			**Responses**	
		Yes	No	Can’t tell	Comments
Screening questions (for all types)	S1. Are there clear research questions?	x			
	S2. Do the collected data allow to address the research questions?	x			
	*Further appraisal may not be feasible or appropriate when the answer is ‘No’ or ‘Can’t tell’ to one or both screening questions*.				
1. Qualitative	1.1. Is the qualitative approach appropriate to answer the research question?	x			
	1.2. Are the qualitative data collection methods adequate to address the research question?	x			
	1.3. Are the findings adequately derived from the data?	x			
	1.4. Is the interpretation of results sufficiently substantiated by data?	x			
	1.5. Is there coherence between qualitative data sources, collection, analysis and interpretation?	x			
**Study: May et al**. [71]					
**Category of study designs**	**Methodological quality criteria**			**Responses**	
		Yes	No	Can’t tell	Comments
Screening questions (for all types)	S1. Are there clear research questions?	x			
	S2. Do the collected data allow to address the research questions?	x			
	*Further appraisal may not be feasible or appropriate when the answer is ‘No’ or ‘Can’t tell’ to one or both screening questions*.				
5. Mixed methods	5.1. Is there an adequate rationale for using a mixed methods design to address the research question?	x			
	5.2. Are the different components of the study effectively integrated to answer the research question?	x			
	5.3. Are the outputs of the integration of qualitative and quantitative components adequately interpreted?	x			
	5.4. Are divergences and inconsistencies between quantitative and qualitative results adequately addressed?	x			
	5.5. Do the different components of the study adhere to the quality criteria of each tradition of the methods involved?	x			
**Study: Rozenblum et al**. [72]					
**Category of study designs**	**Methodological quality criteria**			**Responses**	
		Yes	No	Can’t tell	Comments
Screening questions (for all types)	S1. Are there clear research questions?	x			
	S2. Do the collected data allow to address the research questions?	x			
	*Further appraisal may not be feasible or appropriate when the answer is ‘No’ or ‘Can’t tell’ to one or both screening questions*.				
1. Qualitative	1.1. Is the qualitative approach appropriate to answer the research question?	x			
	1.2. Are the qualitative data collection methods adequate to address the research question?	x			
	1.3. Are the findings adequately derived from the data?	x			
	1.4. Is the interpretation of results sufficiently substantiated by data?	x			
	1.5. Is there coherence between qualitative data sources, collection, analysis and interpretation?	x			
**Study: Sherwood et al**. [73]					
**Category of study designs**	**Methodological quality criteria**			**Responses**	
		Yes	No	Can’t tell	Comments
Screening questions (for all types)	S1. Are there clear research questions?	x			
	S2. Do the collected data allow to address the research questions?	x			
	*Further appraisal may not be feasible or appropriate when the answer is ‘No’ or ‘Can’t tell’ to one or both screening questions*.				
2. Quantitative randomised controlled trials	2.1. Is randomisation appropriately performed?	X			Pilot RCT
	2.2. Are the groups comparable at baseline?	x			
	2.3. Are there complete outcome data?	X			Reasonably
	2.4. Are outcome assessors blinded to the intervention provided?			x	Not mentioned
	2.5 Did the participants adhere to the assigned intervention?	X			Reasonably
**Study: Simpson et al**. [74]					
**Category of study designs**	**Methodological quality criteria**			**Responses**	
		Yes	No	Can’t tell	Comments
Screening questions (for all types)	S1. Are there clear research questions?	x			
	S2. Do the collected data allow to address the research questions?	x			
	*Further appraisal may not be feasible or appropriate when the answer is ‘No’ or ‘Can’t tell’ to one or both screening questions*.				
2. Quantitative randomised controlled trials	2.1. Is randomisation appropriately performed?	X			Feasibility RCT
	2.2. Are the groups comparable at baseline?	x			
	2.3. Are there complete outcome data?	X			Reasonably
	2.4. Are outcome assessors blinded to the intervention provided?		X		
	2.5 Did the participants adhere to the assigned intervention?	X			Reasonably
**Study: Unick et al**. [75]					
**Category of study designs**	**Methodological quality criteria**			**Responses**	
		Yes	No	Can’t tell	Comments
Screening questions (for all types)	S1. Are there clear research questions?	x			
	S2. Do the collected data allow to address the research questions?	x			
	*Further appraisal may not be feasible or appropriate when the answer is ‘No’ or ‘Can’t tell’ to one or both screening questions*.				
4. Quantitative descriptive	4.1. Is the sampling strategy relevant to address the research question?	x			
	4.2. Is the sample representative of the target population?	x			
	4.3. Are the measurements appropriate?	x			
	4.4. Is the risk of nonresponse bias low?	X			Reasonably
	4.5. Is the statistical analysis appropriate to answer the research question?	x			
**Study: Van Buerden et al**. [76]					
**Category of study designs**	**Methodological quality criteria**			**Responses**	
		Yes	No	Can’t tell	Comments
Screening questions (for all types)	S1. Are there clear research questions?	x			
	S2. Do the collected data allow to address the research questions?	x			
	*Further appraisal may not be feasible or appropriate when the answer is ‘No’ or ‘Can’t tell’ to one or both screening questions*.				
1. Qualitative	1.1. Is the qualitative approach appropriate to answer the research question?	x			
	1.2. Are the qualitative data collection methods adequate to address the research question?	x			
	1.3. Are the findings adequately derived from the data?	x			
	1.4. Is the interpretation of results sufficiently substantiated by data?	x			
	1.5. Is there coherence between qualitative data sources, collection, analysis and interpretation?	x			
**Study: Vaz et al.**[77]					
**Category of study designs**	**Methodological quality criteria**			**Responses**	
		Yes	No	Can’t tell	Comments
Screening questions (for all types)	S1. Are there clear research questions?	x			
	S2. Do the collected data allow to address the research questions?	x			
	*Further appraisal may not be feasible or appropriate when the answer is ‘No’ or ‘Can’t tell’ to one or both screening questions*.				
2. Quantitative randomised controlled trials	2.1. Is randomisation appropriately performed?	x			
	2.2. Are the groups comparable at baseline?	x			
	2.3. Are there complete outcome data?	X			Reasonably
	2.4. Are outcome assessors blinded to the intervention provided?			x	Not mentioned
	2.5 Did the participants adhere to the assigned intervention?	X			Reasonably
**Study: Voils et al**. [78]					
**Category of study designs**	**Methodological quality criteria**			**Responses**	
		Yes	No	Can’t tell	Comments
Screening questions (for all types)	S1. Are there clear research questions?	x			
	S2. Do the collected data allow to address the research questions?	x			
	*Further appraisal may not be feasible or appropriate when the answer is ‘No’ or ‘Can’t tell’ to one or both screening questions*.				
5. Mixed methods	5.1. Is there an adequate rationale for using a mixed methods design to address the research question?	x			
	5.2. Are the different components of the study effectively integrated to answer the research question?	x			
	5.3. Are the outputs of the integration of qualitative and quantitative components adequately interpreted?	x			
	5.4. Are divergences and inconsistencies between quantitative and qualitative results adequately addressed?	x			
	5.5. Do the different components of the study adhere to the quality criteria of each tradition of the methods involved?	x			
**Study: Waring et al**. [79]					
**Category of study designs**	**Methodological quality criteria**			**Responses**	
		Yes	No	Can’t tell	Comments
Screening questions (for all types)	S1. Are there clear research questions?	x			
	S2. Do the collected data allow to address the research questions?	x			
	*Further appraisal may not be feasible or appropriate when the answer is ‘No’ or ‘Can’t tell’ to one or both screening questions*.				
4. Quantitative descriptive	4.1. Is the sampling strategy relevant to address the research question?	x			
	4.2. Is the sample representative of the target population?	x			
	4.3. Are the measurements appropriate?	x			
	4.4. Is the risk of nonresponse bias low?	X			Reasonably
	4.5. Is the statistical analysis appropriate to answer the research question?	x			
**Study: West et al**. [80]					
**Category of study designs**	**Methodological quality criteria**			**Responses**	
		Yes	No	Can’t tell	Comments
Screening questions (for all types)	S1. Are there clear research questions?	x			
	S2. Do the collected data allow to address the research questions?	x			
	*Further appraisal may not be feasible or appropriate when the answer is ‘No’ or ‘Can’t tell’ to one or both screening questions*.				
2. Quantitative randomised controlled trials	2.1. Is randomisation appropriately performed?	x			
	2.2. Are the groups comparable at baseline?	x			
	2.3. Are there complete outcome data?	X			Reasonably
	2.4. Are outcome assessors blinded to the intervention provided?	x			
	2.5 Did the participants adhere to the assigned intervention?	X			Reasonably

## Results

The search identified 36,561 titles and abstracts. After removing duplicates, the remaining 29,036 titles and abstracts were screened for potential inclusion. Following initial screening there were 656 full-texts reviewed, which identified a total of 39 studies [42–80] which met the inclusion criteria (see Fig. 1).

### Study characteristics

Fifteen of the included studies (41%) were randomised controlled trials [44, 50, 56, 57, 59, 61–67, 74, 77, 80] with the other quantitative designs including non-randomised experimental [60, 69], pre-post [67, 75, 78, 79], cohort, cross-sectional [42, 43, 71] and feasibility/pilot studies [45, 47, 49, 52–54, 58]. Seven studies (19%) utilised only qualitative designs, including focus groups [70, 72], interviews [51, 55, 76], and a mixture of both [46, 48]. Seventy per cent (*n* = 27) of all studies were conducted in the United States [43–45, 47–50, 54, 56–64, 66, 67, 71–73, 75, 77–80] with the remaining undertaken in England [42, 65, 76], Australia [67, 70], Norway [46, 55], Scotland [52, 74], Wales [51], Finland [69] and Switzerland [53]. Within the studies, the intervention sample sizes ranged from 10 to 2818. The mean age ranged from 31.5 to 72.9 years. Intervention studies mostly included participants with a mean age between 40 and 49 years (*n* = 14) [42, 44, 56, 59, 62–64, 68, 69, 73–75, 77, 80] and 50 and 64 years (*n* = 12) [45, 50, 54, 57, 58, 60, 61, 65–67, 78] with a few studies targeting younger (mean age 20–40 years) [79] and older (mean age of >65 years) cohorts [47, 49, 52].

Participants’ ethnicity varied across studies; however, it is important to note that ethnicity was not reported for eleven (30%) of the articles included [46, 48, 52, 55–57, 63, 69, 70, 76]. Of the studies that did report ethnicity, only eight were identified as having an ethnically diverse sample, defined as 50% or more of the whole sample as being from an ethnic minority group [43, 50, 51, 58, 64, 68, 72, 77]. Most studies included all adults regardless of sex; however, several studies specifically targeted females [45, 67, 71, 79, 80] and males [65]. Ten studies reported employment status, with five of these revealing the sample had an employment rate of less than 70% [50, 51, 55, 67, 78]. Twenty-four studies reported educational attainment, with two studies having a sample where less than 20% had a college degree [50, 59].

The ‘remote’ intervention modes of delivery varied across all of the studies which included the use of individual/group-based telephone calls [50, 54, 57, 58, 60, 61, 67, 68, 73, 78], video-conference calls [42, 47–49, 51, 56, 59, 62, 63, 66, 69, 70, 80], interactive websites [52, 53, 63, 71, 72, 74–76, 81], mHealth smartphone applications [43, 44, 50, 66, 74, 77], social networking sites [45, 79], emails [62, 63, 65], online chat messaging/web-forums [55, 62–65] and personalised/interactive text messaging [61, 67, 68, 70, 77, 80]. Many studies delivered the intervention through a combination of modes such as telephone calls alongside personalised text messaging [61, 67, 68, 70]. Some studies delivered the intervention through mHealth smartphone applications with the addition of telephone calls [50], video-conference calls [66], text messaging [77] or interactive websites [74]. Sixteen (43%) studies incorporated a remote monitoring component as part of the intervention [44, 45, 47–49, 52, 54, 56, 59, 60, 66, 72, 74, 75, 77, 80], including wearable technology to self-monitor physical activity [44, 45, 47, 49, 50, 52, 59, 72, 75, 77] alongside platforms to record and self-monitor diet, weight [44, 54, 56, 66, 72, 75, 77] and *‘lifestyle’* behaviours [59]. Several studies included telehealth remote monitoring [48, 60] or WIFI-enabled smart scales [50, 61, 77, 80] which exchanged data synchronously to a third party for monitoring. All intervention and population characteristics are presented in Table 2.

**Table 2:** Study intervention characteristics.

Authors, year country, design	Target population	Sample size, age, sex *N* (%)	Ethnicity, education, employment (%)	Type of technology, intervention description and comparison overview	Engagement (recruitment, retention, and use)
Abbott et al., 2020 [42] England Prospective cross-sectional	Adults with a body mass index (BMI) ≥ 40 kg/m^2^ or ≥35 kg/m^2^ with comorbidities	*N:* 227 A: 44 ± 12 years F: 160 (71) M: /	White British (63) Other (24) Unknown (14)	Video-conference INT: Tier 3 weight management programme. 6 ×1 h online video conference group sessions for 6 months	Recruitment rate of those eligible (*n* = 277/315; 72.1%). Patients who were older and/or from ethnically diverse backgrounds were more likely to decline. The most common reason was lack of internet access and/or lack of digital skills (89.8%, *n* = 79/88) with 10.2% (*n* = 9/88) who declined as they preferred face-to-face sessions.
Abshire et al., 2020 [43] United States Cross-sectional survey	Primary care patients with a BMI ≥ 30 kg	*N* = 77 A: 46 ± 13 years F: 72 (94) M: 5 (6)	African American (67) White (22) Mixed (7) Other (4) <High school (9) High school (16) College (36) >degree (40)	n/a	n/a
Allen et al., 2013 [44] United States RCT	Individuals aged 21–65 years with a BMI of 28–42 kg/m^2^ with access to a smartphone	*N* = 68 INT: (*n* = 16); CON (1) (*n* = 18) CON (2) (*n* = 17) CON (3) (*n* = 17). A: 44.9 ± 11.1 years F: 53 (78) M: 15 (22)	African American (49) College (67) Fulltime employment (84)	mHealth App + in-person counselling INT: Months 2–6: biweekly 1 x1 h counselling session for 6 months delivered by nutritionist coach. Weight loss mHealth app which provides real-time feedback, self-monitors food intake/activity, and tracks progress with weekly weigh-ins encouraged with opportunities for social networking and support. COM: (1) intensive counselling (2) less intensive counselling + smartphone App (3) Smartphone App only	Recruitment rate of those eligible (*n* = 68/110; 62%). Refusal is mostly related to inconvenience. 63% Follow-up (*n* = 43). Counselling sessions attended (*M* = 72%) days of diet smartphone entries (*M* = 53%); days of physical activity smartphone entries (*M* = 32%)
Arigo et al., 2015 [45] United States Feasibility study	Women aged ≥25 years engaging in less than 30 min of moderate-vigorous activity per week	*N* = 20 A: 50 ± 7.2 years F: 20 (100) M: n/a	White (95) Degree (70)	Hybrid face-to-face/online delivery through social network INT: 1×90-min face-to-face initial skills session delivered by the research team. Encouraged to contribute 1 post per week to the forum. Dashboard to check daily Physical activity totals, encouraged to sync devices & check progress at least twice per day. Intervention lasted 6 weeks.	Recruitment rate of those eligible (*n* = 20/31; 65%). 1 was injured, 6 had no response and 3 declined after receiving an overview of the study. High retention (100%). High engagement with participants wearing trackers on 97% of days on average (range 80–100%).
Asbjornsen et al., 2020 [46] Norway Qualitative interviews and focus groups	Adults aged ≥18 years with a BMI ≥ 30 kg/m^2^.	*N* = 23 A: 53 (24–70) years F: 14 (67) M: 9 (33)	/	n/a	n/a
Batsis et al., 2019 [47] United States Feasibility pre-post study	Adults aged ≥65 years with BMI ≥ 30 kg/m^2^ who have access to high-speed internet.	*N* = 37 A: 69 ± 11.6 years F: 32 (87) M: /	White (100)	Video-conference INT: 16 x weekly 1-1 sessions via video calls delivered by health coach, registered dietitian, & nurse exercise specialist.	Recruitment rate of those eligible (37/58; 64%). Reason for decline related to timing/logistical reasons (*n* = 11) and uninterest to participate in a video-delivered intervention (*n* = 10). Retention was moderate 76% (27/37). Discontinuation was mostly related to noncompliance; one participant’s discontinuation was due to technology issues. Approximately 93%, 96%, and 67% of participants attended greater than 75% of health coach, nurse, and dietitian sessions, respectively.
Batsis et al., 2020 [48] United States Qualitative interviews and focus groups	Adults aged ≥65 years who live in a rural location with a BMI ≥ 30 kg/m^2^.	*N* = 29 A: 72.9 years F: 16 (55) M: /	/	Video-conference INT: Weekly x 1-1 counselling sessions (15–20 min) delivered by a dietician, (Total = 26) and biweekly group exercise sessions (70–90 min) (Total = 13 sessions) delivered by an exercise instructor over 26 weeks. COM: n/a	n/a
Batsis et al., 2021 [49] United States Feasibility pre-post study	Adults aged ≥65 years who live in a rural location with a BMI ≥ 30 kg/m^2^.	*N* = 53 A: 72.9 ± 3.9 yrs F: 37 (70) M: /	High school (13.2) College degree (28.3) Post-college degree (30.2)	Video-conference INT: 18 × 30 min1-1 live videoconference nutrition sessions delivered by registered dietician nurse + 7 1 x h group sessions (remote if necessary) + 75 min x 2 weekly synchronous videoconferencing group exercise sessions delivered by personal trainer (Total = 40 sessions). Every 3–4 weeks an additional on-site group session. Weekly food records reviewed, and attendance monitored. Activity tracker to self-monitor physical activity. Intervention lasted 26 weeks.	Recruitment rate of those eligible (53 /115; 46%). Reason for decline related to being uninterested in participating in a video-delivered intervention (*n* = 27) worry about technology (*n* = 5) and logistical/competing responsibilities (*n* = 30). High retention was found for the intervention arm (44/53; 83.0%). Attendance rates for video/on-site visits were 77% and 78.2% for exercise sessions and 84% and 90.0%, for dietician visits respectively. Participants wore wearable trackers for an average of 81.7% days with a mean time of 8.3 ± 3.8 h per day.
Bennett et al., 2018 [50] United States RCT	Filipino adults with a BMI of >23 kg/m^2^ diagnosed with Type 2 Diabetes (non-insulin-dependent) and own a smartphone, tablet, or laptop computer	*N* = 351 INT (*n* = 176) CON (*n* = 175) A: 50.9 ± 9.1 years F: 120 (68) M: 112 (32)	Non-Hispanic black (52) Non-Hispanic white (29) Hispanic (13) Other/missing (5) >High school graduate (15) High school graduate (36) Some college (40) >College degree (10) Employed (67)	mHealth App and telephone calls INT: The intervention included 1) tailored behavioural goals (e.g., walk 10,000 steps/day, no sugary drinks, no fast food); 2) self-monitoring of these goals via weekly interactive voice response phone calls/text messages; 3) daily self-weighing via a cellular-connected scale; 4) skills training materials in print and video; 5) 18 weight loss counselling coaching calls with a registered dietitian; and 6) brief weight loss counselling at medical visits. Intervention lasted 12 months. COM: Usual Care	Recruitment rate not reported. High retention in the intervention arm (96%) and usual care group (92%) at 12-month visits. 90% completed all three study visits. Intervention participants completed a median of 93.2% and 89% of weekly self-monitoring and coaching calls respectively. Participants weighed for 42.9% of the expected days.
Cliffe et al., 2021 [51] Wales Qualitative interviews	Adults with a BMI of 35–45 kg/m^2^ are referred by healthcare professionals to a dietetics service.	*N* = 13 A: 48.5 ± 20.2 years F: 8 (62) M: 5 (39)	White British (85) White European (15) Employed (54) Retired (23) Student (15) Carer (8)	Video-conference INT: Months 1–2: weekly group sessions; Months 4 + 6 group review sessions (Total = 10) delivered via a registered dietician. Written information and visual materials were sent via email a week before each session. Materials with hands-on activities. Intervention lasted 6 months. COM: Usual Care	Recruitment rate of those eligible (14 /89; 17%). Reason for decline related to no response (42; 47%) and opting for face-to-face sessions (33; 37%). Retention was moderate: 71% (10/14). Two withdrawals due to poor internet connection with one due to a medical condition. *n* = 12 attended first session and *n* = 10 attended >5 sessions (83%).
Connelly et al., 2017 [52] Scotland Feasibility RCT	Adults with Type 2 Diabetes living in remote or rural locations	*N* = 31 Interactive web (*n* = 11) Information web (*n* = 10); CON (*n* = 10). A: 67.3 ± 10.4 years F: 13 (41) M: 18 (59)	/	Interactive website INT: Interactive web group with online access to diabetes-specific physical activity information and interactive features. Intervention lasted 6 months.	Recruitment rate of those eligible/interested in taking part (31/42; 74%). Retention rate was high (90%). <3 months participants logged an average of 12.5 (SD 15.7) times dropping to 11.3 (SD 37.1) times from 3- to 6-month follow-up. There was a large range in the number of logins per month (0–50). In the last 2 months, only 1 person continued to use the website.
Cook et al., 2019 [53] Switzerland Feasibility pre-post study	Adults aged 35 to 65 years with a BMI of 30–39 kg/m^2^	*N* = 23 A: 49.7 years F: 16 (70) M: 7 (30)	Caucasian (78) Hispanic (17) Mixed (4)	Interactive website INT: Online Bulletin Board (OBB) website logins twice daily for at least 30 min for four consecutive days followed by 2 × 1.5 h telephone group discussions.	n/a
Damschroder et al., 2010 [54] United States Pilot feasibility pre-post study	Veterans that were eligible for the MOVE! Lifestyle programme.	*N* = 14 A: 53.8 ± 12.5 years F: 5 (36) M: 9 (64)	Minority ethnic groups (14)	Telephone calls INT: Week 1 (1-1 face-to-face sessions); Weeks 2–5 (Weekly phone calls); Week 5 (onsite visit with a coach; Weeks 6–11 (weekly phone calls); Week 12 face to face. All sessions were delivered by a lifestyle coach. Activity tracker to self-monitor physical activity.	Recruitment rate (15/29; 52%). 11 declined to participate mostly related to not wanting to take part in a weight management study/preferring a group-based programme. High retention (100%). An average of 1.7 phone call attempts were made for each of the 146 completed coaching. The average duration of phone sessions was 32.4 (7.6) min. 92% of the 168 planned sessions (14 participants 12 sessions) were completed.
Das et al., 2014 [55] Norway Qualitative case study incorporating forum extracts and interviews	Adults with basic proficiency in Norwegian enrolled in a bariatric weight loss programme.	*N* = 60 A: 40 ± 9.3 years F: 45 (75) M: /	Primary school (7) High school (53) University/college (40) Employed (66) Student (5) Unable to work/unemployed (27)	Online forum INT: Online discussion forum and personal one-to-one communication (patient-to-patient and health care professional-to-patient or vice-versa). The moderator (researcher) posted weekly topics relevant to the patient group. 5 healthcare professionals *N* = 5) (1 x psychiatric nurse, 1x head nurse, 2 x nurses, 1 x dietician) had access to the eHealth portal and responded to any requests.	n/a
Das et al., 2017 [56] United States RCT	Adults already enrolled on a commercial weight loss programme.	*N* = 644 A: 18–39 (19), 40–59 (56), 60+ (25) years F: 563 (87) M: 81 (69	/	Video-conference INT: 11 x weekly 1-h group meetings (either in community or worksite settings). Participants communicate with the group leader/other participants via a website message board and are encouraged to log weight. COM: Same intervention but delivered in person	Recruitment rate not reported/unknown. There were 71.6% complete reporters (461/644) defined as reporting at least one weight during the final week of their 11-week programme. Videoconference participants, older adults, and enrollees in incentivised programmes were more likely to be a complete reporters.
Donnelly et al., 2007 [57] United States RCT	Adults with a BMI ≥ 30 kg/m^2^ are otherwise judged as healthy and not using tobacco products.	*N* = 97 INT (*n* = 34) clinic (*n* = 39) CON (*n* = 24) A: 53 ± 42 years F: 51 (53) M: 23 (24) Missing: 23 (23)	/	Group phone call INT: Weekly 60-min group phone calls with a health educator for 26 weeks. COM: Face-to-face clinic	Recruitment rate not reported/unknown. Retention at 12 weeks was moderate (74/97; 76%). Nine participants (26%) terminated/withdrew from the phone group, 12 from the clinic (31%), and two from control groups (9%). Reasons for attrition included not meeting the attendance requirement (scheduling conflicts), noncompliance with the study protocol, dissatisfaction with study conditions, and the participant’s perception they could continue successfully on their own.
Dutton et al., 2015 [58] United States Pilot feasibility pre-post study	Adults with a BMI ≥ 30 kg/m^2^ with ≥1 cardiometabolic risk factor/s.	*N* = 33 A: 56 ± 10.2 years F: 29 (88) M: 4 (12)	Black African American (85) ≤High school (42.4)	Hybrid face-to-face/telephone calls INT: 12 × 60 min group-based office visits (with private weigh-ins) delivered by health professionals plus 12 individual phone contacts over 6 months (15 min) delivered by trained peer coaches.	Recruitment rate not reported/unknown. High retention (85%). Participants attended approx. 50% of the group visits (6 ± 4 of 12 possible sessions) and completed approximately 40% of the intended telephone calls (i.e., 5 ± 3 of 12 scheduled calls). The mean duration of completed calls was 14 ± 7 min.
Gibson et al., 2020 [59] United States RCT with mixed methods evaluation	Adult kidney transplant recipients with a BMI > 22 kg/m^2^ and the ability to report data with internet access.	*N* = 10 INT (*n* = 5) CON (*n* = 5) A: 45.2 ± 10.2 years F: 5 (50) M: 5 (50)	Non-Hispanic white (50) Black African (20) Mixed (20) Hispanic (10) High school (40) Some college (40) College graduate (20)	Video-conference INT: 12 weeks of weekly 1-h group-based health coaching sessions delivered remotely by a registered dietitian and physical activity expert followed by 12 weeks of maintaining healthy behaviours. Participants were encouraged to report healthy lifestyle behaviours weekly and to accumulate at least 150 min of moderate-intensity PA per week. Intervention lasted 6 months.	Recruitment rate of those eligible (10/10; 100%). High retention: 9/10 (90%) with one voluntary withdrawal although not related to the intervention. The attendance rate of health coaching sessions was 78% for the 12 sessions. Absences related to illness or schedule conflicts. Adherence to reporting healthy behaviours was 86% with technological issues cited as barriers to full reporting.
Goodrich et al., 2018 [60] United States Non-randomised trial with mixed methods evaluation	Veterans screened as having a BMI ≥ 25	*N* = 2818 INT (*n* = 530) CON (*n* = 2282) A: 57 ± 9.5 years F: 75 (15) M: 422 (85) 42 interviews with key stakeholders (dieticians, physicians, co-ordinators, champions)	White (80) Black (16) Other (3)	Telephone calls INT: Participants receive an Interactive TeleMOVE messaging device alongside a Health Buddy, MOVE! handout booklet, a pedometer to track daily activity and a home-based digital scale. In the course of these interactive dialogues with the health buddy, patients entered weight information and any responses to daily prompts to be forwarded via landline phone each night to a vendor server which is forwarded to a co-ordinator. Participants also received 10- to 20-min telephone calls from a TeleMOVE coordinator every 30 days for any red alert questions.	Recruitment rate not reported/unknown. High retention of 2 or more visits (6 months) in the intervention arm TeleMOVE (93.9%; 467/497) compared to the control arm MOVE! (71.97%; 1189/1652). Less engaged from non-white backgrounds in TeleMOVE (20%) compared to tMOVE! (35%). More people who live in a rural location engaged in TeleMOVE (57.9%) compared to MOVE! (41.93%).
Haggerty et al., 2016 [61] United States RCT/ Survey	Women with a BMI ≥ 30 kg/m^2^ and endometrial hyperplasia or Type I endometrial cancer.	*N* = 20 Telemedicine (*n* = 10) Texting (*n* = 10) A: Median 63 years F: 20 (100) M: n/a	White (68) Black (25) Asian (4) Other (3)	Telephone calls + text messages. INT: 16 weekly telephone counselling sessions followed by bi-weekly sessions (weeks 18–24) delivered by two interventionists (master’s level clinician and medical doctor). Weight was recorded via a Wi-Fi scale shared via an internet platform with real-time feedback on participants’ progress.	Recruitment rate (60% of women approached expressed interest in participation; around 50% were eligible based on technology capabilities). High retention. High retention (100%) and adherence (100%).
Harvey-Berino et al., 2002 [62] United States RCT	Adults with a BMI ≥ 25 kg/m^2^ and access to a computer/internet.	*N* = 124 Internet support (*n* = 40) Minimal in person support (*n* = 41) Frequent in person support (*n* = 41) A: Intervention only 46.3 ± 11.1 years F: 36 (90) - M: 4 (10)	White (98) High school (10) Some college (25) College degree (28) Graduate (38)	Email, video chat + discussion groups INT: 1-h weekly sessions (*N* = 24) delivered by therapist for 24 weeks. COM: (1) Frequent in-person support. (2) Minimal in-person support	Recruitment rate not reported/unknown. Attrition was 18% after 6 months of treatment and 24% over 18 months of evaluation. A total retention rate of 73% for all data points. Attendance was greater for face-to-face conditions than for the internet support condition.
Harvey-Berino et al., 2004 [63] United States RCT	Adults with a BMI ≥ 25 kg/m^2^) and access to a computer/internet.	*N* = 255 Internet (*n* = 77) Minimal in person support (*n* = 78) Frequent in person support (*n* = 77) A: 45.8 ± 8.9 years F: 209 (82) M: 46 (18)	High school (9) Some college (29) College degree (29) Graduate school (33)	Interactive television, email, + web chat INT: 1-h weekly sessions (*N* = 24) delivered by local health educators, dietitians, site facilitators and a dietitian for 24 weeks. COM: (1) Frequent in-person support (2) Minimal in-person support	Recruitment rate not reported/unknown. Retention was highest for the frequent in-person and minimal in-person with 88% (68/77) and 86% (67/78; 86%) compared to the internet support group (77%; 59/77) at 12 months. Participants in the face-to-face condition attended significantly more group meetings than those in the internet support condition. Participants in the internet condition submitted self-monitoring diaries more frequently and reported significantly more peer support contacts than those in the face-to-face condition.
Harvey-Berino et al., 2010 [64] United States RCT	Adults with a BMI between 25 and 50 kg/m^2^ and access to a computer/internet.	*N* = 481 Internet (*n* = 161) Hybrid (*n* = 162) In person (*n* = 158) A: 46.6 ± 9.9 years F: 447 (93) M: 34 (7)	African American (66) College graduate (65)	Synchronous online chat INT: 1-h weekly sessions (*N* = 24) delivered by graduate students, clinical psychologists, and registered dieticians for 24 weeks. COM: (1) In-person (2) Hybrid (Internet + in-person)	Invited/consent rate (658/1143; 56%). Declines mostly related to loss of interest (171;485; 35%) and no-show (293/485; 60.41%). The Internet group had the highest retention rate (159/161; 99%) although retention was high across all groups (in person: 150/158; 95%, hybrid: 153/162; 94%). There were no significant differences in group sessions attended across conditions (76% Internet vs. 71% in-person vs. 72% Hybrid) or in the proportion of self-monitoring journals submitted.
Haste et al., 2017 [65] England RCT with mixed methods evaluation	Men with Type 2 diabetes and a BMI between 30–40 kg/m^2^.	*N* = 61 INT (*n* = 33) CON (*n* = 28) A: 58 years F: n/a M: 61 (100)	White (100) *M* = 12 years of education Employed (83) Retired (41) Unemployed (15) Unable to work (6)	Personalised web-based email-style consultations INT: Dietician-based consultations: Months 1–3: weekly web-based sessions (Total = 12) Months 4–12 (monthly web-based sessions (Total = 9). Exercise expert-based consultations: Months 1–3: Monthly web-based sessions (Total = 3), Months 4–12: Quarterly web-based sessions (Total = 3) delivered by dietitian and an exercise expert. Intervention lasted 12 months. COM: Usual care	Invited/consent rate (61/968; 6.3%). This is mostly related to decline to participate (187/907; 21%) and non-response (696/907; 77%). Retention was higher in the intervention group at 3 months (INT: 73%, CON: 57%) and at 12 months (INT: 61%, CON: 43%). Logins to website at 12 months was a median of 43 (12–167), higher food intake entries with a median of 99 (3–246) compared to exercise entries 262 (0–262).
Hu et al., 2021 [66] United States RCT/ Survey	Adults (18–80 years) living with excess weight and prediabetic OR diagnosed with Type 2 diabetes.	*N* = 161 INT (*n* = 84) CON (*n* = 77) A: 58.6 ± 11.1 years F: 107 (67) M: 54 (34)	White (55) Black African (25) Other (19) Missing (1) High school (17) Foundation degree (13) >Bachelor’s degree (70) Employed (72)	Video-conference + mHealth App INT: Month 1: 1 x weekly sessions; Months 2–6: 1 x biweekly sessions (*N* = 14 sessions) delivered by the research team (dietitian, research associate). Intervention lasted 6 months.	Recruitment rate not reported/unknown. Mean WebEx attendance = intervention 74.4% control 74.3%. Days participants logged daily calorie goal in App (INT: 45.1; CON: 27.5). 84% completed post-study questionnaire (*n* = 100).
Job et al., 2017 [67] Australia RCT with mixed methods evaluation	Women (18–75 years) diagnosed with stage I–III breast cancer with BMI 25–40 kg/m^2^	*N* = 45 A: 56.0 ± 12.0 F: 45 (100) M: n/a	White (96) >High school (73) Employed (62)	Phone calls + text messages INT: 16 phone calls delivered by an accredited dietitian with an extended 6-month phase with tailored text messages delivered. Intervention lasted 12 months. COM: Usual care	Recruitment rate in intervention of those eligible (30/40; 75%). Did not own a phone (*n* = 3), did not need support (*n* = 1), family/health reasons (*n* = 3), used phone for emergencies only (*n* = 1) with 2 not contactable. More than half (57%, 17/30) of the women who participated in the extended contact intervention had received text messages during the initial 6-month intervention.
Kolodziejczyk et al., 2013 [68] United States Pre-post study	Adults (21–60 years) with a BMI of 27.0–39.9 and willing/able to learn to text and can communicate in English/Spanish.	*N* = 20 A: 40.10 ± 8.05 years F: 12 (60) M: /	Hispanic (75) White non-Hispanic (65) Asian (10) Missing (20) College (40) College graduate (15) Graduate degree (35) Missing (10)	Phone calls + text messages INT: 3–5 x daily automatically scheduled and tailored text messages encouragement and reinforcement delivered by the research team. Intervention lasted 8 Weeks	Recruitment rate not reported/unknown. High retention rate (90%; *n* = 18). Participants responded to 88.04% (986/1120) of interactive text messages.
Latinen et al., 2010 [69] Finland Non-randomised trial with mixed methods evaluation	Adults at high risk of Type 2 Diabetes	*N* = 74 INT (*n* = 33) CON (*n* = 41) A: 49 years F: 41 (55) M:33 (65)	/	Video-conference INT: 90-min group sessions at 2-week intervals with session 5 at 6 months delivered by a clinical nutritionist. Intervention lasted 6 months. COM: The same intervention delivered face-to-face	Recruitment rate not reported/unknown. High retention (73/74; 99%).
Lewis et al., 2021 [70] Australia Qualitative Focus groups	Participants who completed a previous telehealth trial	*N* = 15 A: 55 ± 12.0 years F: 12 (80) M: 3 (20)	/	Video-conference + text message INT: 3 texts + 1 phone call per week x 4 months delivered by an accredited practising dietitian and exercise physiologist specialising in obesity. Intervention lasted 4 months.	n/a
May et al., 2019 [71] United States Cross-sectional survey	Female cancer survivors who completed active cancer treatment and are living with excess weight.	*N* = 96 A: 54.3 ± 9.6 years F: 96 (100)	White (89) Black (4) Asian (3) Hispanic (4) Native American (1) Grades 0–12 (2) College (26) College graduate (28) >Degree (44)	Interactive website	n/a
Rozenblum et al., 2019 [72] United States Qualitative Focus groups	Adults (20–70 years) with a BMI of 27–35 kg/m^2^ with internet access.	*N* = 13 A: Aged 20–70 F: 10 (77) M: 3 (23)	African American (46) White (31) Other (8) Missing (15) >8th Grade (8) High school (15) College (54) >Degree (23)	Interactive website	n/a
Sherwood et al., 2010 [73] United States RCT	Adults with a BMI between 30 and 39 kg/m^2^ free of any health conditions & not participating in a weight loss programme	*N* = 63 INT 10 calls (*n* = 21) INT 20 calls (*n* = 21) CON (*n* = 21) A: 49.5 ± 2.5 F: 50 (79) M: 23 (21)	White (83) College/ degree (58) Professional (46) Clerical/labour/other (54)	Telephone calls INT: 20 × 10–20 min telephone calls delivered by a counsellor. Intervention lasted 10 Weeks. COM: (1) Self-directed: received the same instructional manual, pedometer & log booklets but not contacted (2) Same intervention but only 10 (out of 20) contacts	Recruitment rate (63/187; 34%). Reason for decline was related to no contact info (38/124), not interested (7/124) and not eligible (79/124). *Retention for the self-directed*: 16/21; 76%); *10 call group*: (17/21; 81%); 20 call group (18/21; 86%). Sessions completed*:* 0 (*n* = 1), 1–10 (*n* = 8), 11–19 (n = 6), 20 (*n* = 6). Increased contact associated with increased engagement (self-weighing, exercise).
Simpson et al., 2020 [74] Scotland RCT with mixed methods evaluation	Adults with a BMI ≥ 30 kg/m^2^ who own a smartphone.	*N* = 109 INT (*n* = 73) CON (*n* = 36) A: 46.2 ± 10.6 F: 76 (70) M: 33 (30)	White British/Irish (84) White other (6) Indian (2) Pakistani (2) Chinese (1) Higher education (62) Other (38) Employed (94) Unemployed (6)	mHealth App + interactive website INT: Delivered by “Helpers”—People who the participant has nominated to support them. Intervention lasted 12 months. COM: Received leaflet about health benefits of healthy eating/physical activity. No social support or personalised content.	Recruitment rate of those eligible (109/188; 56%). Reason for decline related to not meeting exclusion criteria (32/89), decline/non-response (40/89) and other reasons not specified (7/89). Retention for the intervention group was 77% and the control group 71%. Helper engagement with app was low, whereby only 54 (74%) downloaded the app and 48 (89%) used it twice or more. 28 helpers enrolled via the app, and 19 (36%) participants interacted with their helper(s) via the app.
Unick et al., 2019 [75] United States Pre-post study/Survey	Adults (aged 18–70) with a BMI ≥ 25 kg m^2^ with internet access.	*N* = 119 A: 49.8 ± 9.8 F: 99 (83) M: /	White Non-Hispanic (86)	Interactive website INT: 4 x weekly sessions.	Recruitment rate not reported/unknown. 130 Enrolled, 119 completed with a retention rate of 91.5%. Early non responders (4-week weight loss <2%) viewed 3.6 ± 0.8 with initial responders (4-week weight loss ≥2%) viewing 3.7 ± 0.9. Website logins (15.5 ± 12.4 vs. 18.5 ± 10.5), weight self-monitoring (26.8 ± 2.5 vs. 27.2 ± 2.2) and *n* days calorie intake was reported (26.4 ± 3.2 vs. 27.3 ± 2.4) were similar.
Van Beurden et al., 2018 [76] England Qualitative interviews	Adults (aged 35–60) with a BMI of 30–45 kg/m^2^ and internet access.	*N* = 20 A: 35–37 F: 14 (70) M: 6 (30)	/	Interactive website INT: Primary care + web-based delivered by nutritionists and exercise instructors. Intervention lasted 4 weeks.	n/a
Vaz et al., 2020 [77] United States RCT	Aged (aged 18–65) with a BMI of 25–42 kg/m^2^ employed in a sedentary job with access to a smartphone.	*N* = 28 INT (*n* = 13) CON (*n* = 15) A: 43.25 ± 2.48 F: 24 (86) M: 4 (14)	African American (32) Caucasian (43) Hispanic (18) Asian (7)	mHealth App + text messaging INT: Primary care + web-based. Intervention lasted 6 months. COM: Waitlist-control	Recruitment rate not reported/unknown. 100% retention at 6 months for all participants who did the intervention. All participants engaged in all of the key components. Group chat messages sent was 0.36 ± 0.09 (mean ± SE) per day, *n* food photographs shared 0.41 ± 0.12 (mean ± SE) per day. Participants stepped on a smartscale 0.77 times per day with a daily weight rate of 0.47.
Voils et al., 2020 [78] United States Pre-post study with mixed methods evaluation	Veterans who have had bariatric surgery in the past 12 months	*N* = 30 A: 56.9 ± 10.0 F: 6 (20) M: /	White (73) Black (13) Mixed/other (7) >High school (100) Employed (40) Retired (40) Unemployed (3) Other (17)	Telephone calls INT: Month 1: 4 x weekly calls, Month 2–4: 5 x biweekly calls (*N* = 9 calls), Phone calls addressed behaviour change strategies for diet, physical activity & supplement adherence followed by biweekly phone calls. Intervention lasted 16 weeks.	Recruitment rate of those eligible (33/69; 48%). Reason for decline not provided. Retention at 16-week follow-up was 93% (28/30). 97% (29/30) participated in first telephone call. Mean number of calls received (out of a maximum of 9) was 7.8 (SD = 1.3). Participants who received <9 calls missed calls due to schedule difficulties rather than withdrawal.
Waring et al., 2018 [79] United States Pre-post study	Women (aged ≥18) between 6 weeks and 12 months postpartum, with a BMI of 25–45 kg/m^2^ and access to a scale/smartphone and regularly use Facebook.	*N* = 19 A: 31.5 ± 3.2 F: 19 (100) M: n/a	Non-Hispanic white (74) Non-Hispanic black (5) Hispanic/Latina (11) Asian (11) <Bachelor’s degree (11) Bachelor’s/some graduate school (32) Graduate degree (58) Employed (64) Student (5) Stay-at-home parent (32)	Social networking group INT: A Facebook group delivered by two coaches (a licensed clinical psychologist and a health promotion researcher), an obstetrician and a physical therapist. Intervention lasted 12 weeks.	Recruitment rate of those approached (19/134; 14%). Reason for decline included ineligible (40/115), no contact/decline (64/115), did not complete baseline (10/115) or did not join Facebook group (1/115). High retention (95%; 18/19). Participants posted median of 2 x posts and 24 replies and liked a median of 32 posts or comments. Engagement was sustained through the end of the intervention: 42% of participants posted, commented, or liked a post or comment on the last day of the intervention, 63% during the last week, and 100% in the last 4 weeks.
West et al., 2019 [80] United StatesRCT	Women (aged ≥18 years) with a BMI of 25–50 kg/m^2^ and in good general health	*N* = 32 Videoconference (*n* = 16) Text message (*n* = 16) A: 47.2 ± 12.4 years F: 32 (100) M: n/a	White (78) African American (19) Other (3) Some college <4 years (9) Vocational training (3) College degree (44) Graduate degree (44)	Video-conference /Text messaging INT: 1 x h weekly video-based sessions (*N* = 24 sessions) delivered by an experienced weight control counsellor. Intervention lasted 6 months. COM: None	Recruitment rate of those approached (32/79; 41%). Reason for decline included not meeting inclusion criteria (46/47) and declining to participant (1/47). Moderate retention at 2 (78%) and 6 (75%) months with no significant differences between conditions. More participants withdrew early in the study in the Text group (31%) compared to the Video (12%) group. Video participants attended an average of 15 (62%) chat sessions, and Text participants attended an average of 12 (50%), with no significant difference between groups. Participants in the video condition self-monitored their weight on significantly more days (123 vs. 8 days) and reported physical activity significantly more often compared with those in the Text condition (55 vs. 22 days). No differences found for self-monitoring of dietary intake. Women in the Video condition had significantly greater engagement, with greater self-monitoring and website utilisation than those in the Text condition.

*NB* / data unavailable to extract, *n/a* not applicable.

#### Engagement with a remote weight management intervention

Patterns related to the engagement with remote digital weight loss interventions, classified as the target behaviour for this study, were examined. This focused on the uptake (recruitment to the study/intervention), the duration of usage (retention/adherence) and interaction (automated recorded data including logins and data entry, attendance) with the intervention.

Several studies required participants to be eligible to have access to a high-speed internet connection [47, 59, 62–64, 72, 75, 76, 81, 82] and/or required them to have access to the technology that the intervention was being delivered through, which in the majority of cases was through smartphones [44, 50, 74, 77, 79] and/or laptop/computers (which in some instances required specific operating system requirements) [50, 62–64].

Most studies that reported a recruitment rate (*n* invited/*n* consent) achieved a consent rate of 40% and above. The consent rate was 80% for one study [59]; followed by six studies at 60–79% [42, 44, 45, 47, 52, 59] and seven studies at 40–59% [49, 54, 61, 64, 74, 78, 80]. One study, which was based on delivering weight management sessions via video conference, revealed that older participants and/or those from an ethnic minority group were significantly less likely to consent compared to white younger participants [42]. The most common reason for lack of consent was a lack of interest [45, 47, 49, 64, 73] or perceived need [67] to participate in a remote weight management programme. Inconvenience, alongside competing responsibilities [44, 47, 49], preference for face-to-face support [42, 51, 54], lack of digital skills or technology capability [42, 61], lack of access to the internet [42] or technology, [67] refusing to use their smartphone for the intervention [67] and anxiety about technology [49] were also shown to be barriers to consent. Four studies revealed a consent rate below 40% (0–19% [51, 65, 79], 20–39% [73]). The low levels of consent in these studies, where specified, mostly related to decline (no reason specified) and/or non-response [65, 79]. In one study, 37% declined as they opted for a dietitian-led weight management group through face-to-face sessions rather than synchronously using videoconference [51].

The majority of studies achieved high retention (*n* enrolled/*n* completed), with fourteen studies achieving a retention rate of 90% or above [45, 50, 52, 54, 59–61, 64, 68, 69, 75, 77–79] and four which achieved full retention (100%) [45, 54, 61, 77]. These studies all had interactive components; for example, two were delivered via telephone consultations with the ability to log and track behaviour [54, 61], one study was delivered in hybrid (face-to-face/online) format through a social network website [45], and the other involved a mHealth App alongside personalised text messaging [77]. The inclusion criteria on some studies required participants to have the ability and skills to use the technology, such as sharing data [59] and/or have the willingness to use the technology required as part of the study [68] or already using the platform the intervention was being delivered through (i.e., social networking site) [79]. Where studies used a pre-requisite to either have familiarity or current use of the technology the intervention was being delivered by, there was a trend towards higher retention, with all studies achieving a retention rate of >90%.

Only one study had a retention rate below the 70% threshold [44] (63%). This study had a predominantly ‘*middle-aged’* (*M* = 44.9; SD = 11.1) female (78%) sample with high levels of ethnic diversity (49% African-American). The delivered intervention included intensive biweekly diet and exercise counselling sessions delivered by a nutritionist coach (months 2–6) alongside a weight loss mHealth app, which provided real-time feedback, self-monitored food intake and activity, and tracked progress with weekly weigh-ins encouraged with opportunities for social networking and support. Tracking data identified that intervention usage was highest in the intensive counselling plus smartphone group, with 70% of all sessions attended compared to intensive counselling only (58%).

#### Behavioural diagnosis: COM-B analysis of influences on engagement with remote weight management technologies

The findings revealed a wide range of influences on engagement with remote weight management interventions. When mapped to the COM-B model [32], all constructs in the system were identified via themes related to: psychological capability (*n* = 9); physical capability (*n* = 3); reflective motivation (*n* = 19); automatic motivation (*n* = 11); physical opportunity (*n* = 8); and social opportunity (*n* = 11). The schematic framework is depicted in Table 3. This is explained in more detail below.

**Table 3:** Barriers and facilitators to engagement with remote weight management interventions, deductively mapped to the TDF domains and COM-B constructs.

Thematic barriers and facilitators	Theoretical domains	COM constructs
Sensory (hearing, vision, dexterity) limitations (−) [48] Physical or functional limitations (−) [71]	Skills (physical)	Physical capability
Literacy ability (reading and writing) to use technology (−) [55] Digital competency and technical skills to use technology (+) [42, 45, 48, 60, 66] Familiarity and prior experience of technology (+) [43, 44, 50, 51, 61, 71, 74] Self-monitoring and tracking behaviour (+) [44, 46, 52, 59, 65, 71, 72, 76] Biofeedback (+) [46] Shaping knowledge (+) [46] Tailored feedback (+) [46, 59, 67, 70, 73] Knowledge and skills on how to perform the health behaviour (+) [51, 59, 71] Knowledge and skills on how to set goals (+) [74, 78] Cognitive limitations (i.e. memory loss or forgetfulness) (−) [48, 74]	Knowledge, Skills (cognitive and interpersonal), Memory, attention and decision processes, Behavioural regulation	Psychological capability
Willingness to learn or adopt technology (+) [44, 74] Perceived ease of use and simplicity (+) [44, 48, 49, 60, 65, 66, 70, 72, 74, 76] Perceived usefulness of technology or intervention (+) [47–49, 58, 60, 72] Perceived capability to use technology (+) [61, 62] Perceived confidence to interact with others using technology (+) [61, 67, 71, 74] Autonomy to choose the platform or technology (+) [72] Desire and motivation to lose weight (+) [43, 53, 71] Perceived confidence to change or adapt health behaviour (+) [46, 51, 71, 74, 78] Setting goals and planning (+) [46, 59] Motivational discussions (+) [46, 59, 68] Role and identity (+) [46] Autonomy and personal responsibility (+) [51] Unrealistic expectations (−) [53] Concerns surrounding privacy (−) [55, 57, 71] Preference for face-to-face (−) [42, 51] Perceived time burden to engage in the intervention (−) [45, 59, 65, 71, 78] Perceived performance or effort expectancy (−) [72]	Social/professional role and identity, Beliefs about capabilities, Beliefs about consequences, Optimism, Intentions, Goals	Reflective motivation
Praise through social reward (+) [46] Rewards through material incentives (+) [46, 53] Natural consequences (+) [46] Reduced threat (i.e. less stressful and daunting) (+) [51] Fear of failure (−) [53] User dissatisfaction with technology or intervention (−) [60, 66] Isolation following the end of the intervention (−) [68] Uncomfortable asking others to be helpers in the intervention (−) [74] Embarrassment or anxiety surrounding self-disclosure (−) [45, 48, 55] Anxiety about using technology (−) [49]	Reinforcement, Emotions	Automatic motivation
Convenience (i.e. travel and competing time demands) (+) [48, 51, 52, 56–59, 62, 65, 67, 70, 71, 76, 78, 79] Reduced cost compared to face-to-face (+) [51, 56, 57] Functionality and usability of equipment (+) [49, 60, 65, 72, 74, 76, 79] Opportunity to track, monitor and share behaviour (+) [44, 71, 74, 76] Technical issues (i.e. malfunctions, unresponsive, errors, incompatibility) (−) [51, 59, 60, 66, 68, 69, 74] Access to the specific technology required (−) [48, 49, 51, 52, 56, 60, 65, 67, 72, 76] Access to high-speed internet (−) [43, 48, 51, 61, 65, 66]	Environmental context and resources	Physical opportunity
General social support and engagement (+) [45, 46, 48, 51–53, 55, 57, 59, 61, 70, 71, 73, 76, 78] Peer or group support and encouragement (+) [45, 47, 51, 62, 63, 69, 70, 74, 77, 80] Positive social comparison with peers or others (+) [51, 71] Hybrid interventions with some face-to-face contact or support (+) [42, 43, 47, 54, 60–62, 64, 65, 67] Support from a professional, specialist or healthcare provider (+) [43, 44, 46, 49, 53–55, 65, 68, 74, 78, 80] Positive relationship with the professional (+) [54, 65, 72, 80] Regular check-ins or additional contact and support (+) [44, 53, 54, 58, 60, 65, 67, 72, 74, 78] Accountability (+) [44, 48, 57, 59, 60, 62, 67, 70, 72] Follow-up support (+) [68, 74, 78] Non-judgemental care (+) [70, 72] Emotional support (+) [67, 73]	Social influences	Social opportunity

(+) indicates positive influence (facilitator), (−) indicates negative influence (barrier).

### Physical capability *(TDF domain; skills)*

Physical and/or functional limitations [71] were presented as a barrier which would impact on engagement with a digital intervention in a cross-sectional survey with women who had completed cancer treatment. Findings from a qualitative study with older adults (aged ≥65 years) identified that sensory limitations (e.g., hearing, vision) [48] would impact their ability to engage with a remote intervention.

### Psychological capability (*TDF domains: knowledge; cognitive and interpersonal skills; memory, attention and decision processes; behavioural regulation)*

The knowledge that comes from familiarity and prior experience [43, 44, 50, 51, 61, 71, 74] alongside having the digital competency and technical skills to use the technology [42, 45, 48, 60, 66] were revealed to be important factors that influenced engagement with a remote intervention. Having the acquired knowledge and skills on how to perform a health behaviour [51, 59, 71] and the knowledge and skills on how to set goals and regulate behaviour [74, 78] were also identified as important. Facilitators also included self-monitoring and tracking behaviour [44, 46, 52, 59, 65, 71, 72, 76], shaping knowledge [46] and receiving biological [46] and tailored feedback [46, 59, 67, 70, 73]. Conversely, cognitive limitations (memory loss and forgetfulness) [48, 74], particularly among older populations, negatively impacted how some participants engaged with a remote intervention. Literacy ability (reading and writing) or lack of was shown to influence the use of a remote intervention, reported in one study [55]. In this study, the majority of the sample were only educated to high school level (61%) with around one-third identified as being unemployed.

### Reflective motivation (*TDF domains: beliefs about capabilities; beliefs about consequences; optimism; social/professional role and identity; intentions; goals)*

As with traditionally delivered interventions, the desire and motivation to lose weight [43, 44, 53, 71] alongside the perceived confidence in the capability to change and adapt health behaviour [46, 51, 71, 74, 78] were all found to be important facilitators that influenced engagement of a digital intervention. Some participants declined an intervention as they had a preference for a face-to-face mode of delivery [42, 51] rather than using a remote method. This was particularly noted in studies that included weight management programmes which have been traditionally delivered in person, and/or they were given the option to attend in person. Perceptions related to the ease of use and simplicity [44, 48, 49, 60, 65, 66, 70, 72, 74, 76], perceived usefulness of technology and/or intervention [47–49, 58, 60, 72], perceived capability to use technology [61, 62] and the willingness to learn or adopt the technology [44, 74] were all shown to be important facilitators to engage with a remotely delivered weight loss intervention. Perceived confidence to interact with others using technology [61, 67, 71, 74] was also highlighted as being important. This was particularly noted in interventional studies which used group-based interactive elements (video-conferencing, online forums).

Autonomy to choose the platform and technology that the intervention was delivered on [72] and role and identity [46] were also viewed as a facilitator alongside setting goals and planning [46, 59], having motivational discussions [46, 59, 68], and increased autonomy and personal responsibility [51]. In contrast, several studies revealed that the perceived time burden to engage in the intervention [45, 59, 65, 71, 78] was a barrier to continued engagement; however, this mostly related to interventional studies which requested participants to use daily wearable technology alongside frequent self-monitoring of behaviour. Perceptions related to concerns surrounding the privacy of the technology [55, 57, 71], unrealistic expectations [53] and perceived performance or effort expectancy [72] that was involved in taking part in the intervention were shown to negatively impact engagement.

### Automatic motivation *(TDF domains: reinforcement; emotions)*

Emotional factors, particularly anxiety were revealed as a barrier to engagement in remote interventions. This related to embarrassment or anxiety of self-disclosure, [45, 48, 55] alongside anxiety using technology [49]. In contrast, some participants found remote interventions a reduced threat (less stressful and daunting) when compared to face-to-face interactions [51]. One study required participants to approach others to be their ‘peer’ helper which some participants revealed they felt uncomfortable doing [74]. With facilitators, praise (social reward) [46], rewards (material incentives) [46, 53], and natural consequences [46] were all shown to positively reinforce and influence engagement. User dissatisfaction with technology/intervention [60, 66], fear of failure [53] and isolation following the end of the intervention [68] were all shown to be barriers.

### Physical opportunity *(TDF domains: environmental context and resources)*

The convenience of engaging in the intervention remotely, with less need to travel and less impact on competing demands (e.g. childcare, work) [45, 48, 51, 52, 56–59, 62, 65, 67, 70, 71, 76, 78, 79] alongside reduced cost compared with face-to-face [51, 56, 57] were viewed as the most common facilitators that influenced participants desire to engage with a remote weight-based intervention. Having good levels of functionality and usability of equipment [49, 60, 65, 72, 74, 76, 79], including the opportunity to track, monitor and share behaviour [44, 71, 74, 76] were important facilitators. Technical issues (malfunctions, unresponsive, errors, incompatibility) were revealed to be important barriers that influenced continued engagement in some of the interventions [51, 59, 60, 66, 68, 69, 74]. Many of the interventions required accessing or using specific technology devices or platforms which required particular specifications [48, 49, 51, 52, 56, 60, 65, 67, 72, 76], alongside a high-speed internet connection [43, 48, 51, 61, 65, 66]. Therefore, providing access to these was shown to be integral to consenting to take part in the intervention.

### Social opportunity *(TDF domain: social influences)*

General social support and engagement was identified as one of the most common facilitators [45, 46, 48, 51–53, 55, 57, 59, 61, 70, 71, 73–76, 78], including peer [45, 47, 51, 62, 69, 74] and group support and encouragement [51, 63, 74, 77, 80]. Several studies reported that positive social comparison with peers/others was also viewed as a facilitator for remote interventions [51, 71]. A common facilitator identified among nine studies to engaging with remote interventions was increased accountability [44, 48, 57, 59, 60, 62, 67, 70, 72].

Encouragement and support by a professional or specialist (weight loss/exercise) [43, 44, 46, 49, 53–55, 65, 68, 74, 78, 80] was found to be particularly important. However, a survey conducted with primary care patients living with obesity from an ethnically diverse background viewed encouragement and support with healthcare providers [43] less favourably. Maintaining a positive relationship with the professional [54, 65, 72, 80] alongside the provision of non-judgemental care [70, 72] and emotional support [67, 73] were viewed as important. Hybrid interventions, i.e., those that were delivered remotely but had some face-to-face contact, were also viewed more favourably [42, 43, 47, 54, 60–62, 64, 65, 67], alongside interventions with regular check-ins [44, 53, 54, 58, 60, 65, 67, 72, 74, 78] and follow up support when the intervention has ended were also viewed important [68, 74, 78].

## Discussion

From the 39 articles included in this review, 57 themes were coded; representing 18 barriers and 39 facilitators to remote engagement with weight management interventions. These were then mapped to all six constructs of the COM-B model to provide a detailed ‘behavioural diagnosis’ of areas to optimise and address in future research and practice.

For behaviour to occur, individuals must have the knowledge and skills to engage in or refrain from the desired behaviour [83]. Not having the digital skills to partake in a remote intervention was often noted as a reason for disengagement. In relation to this, even when interventions provided participants with the technology to access the sessions, individuals were still sometimes reluctant to engage due to not knowing how to use the technology [49], which also caused anxiety. Concerns surrounding privacy were also highlighted as a barrier to engagement [55, 57, 71]. Research in other topic areas with remote delivery has also identified that technology anxiety is a barrier to participation when using such methods [84–87]. Therefore, increasing individuals’ knowledge and skills in using technology, including privacy assurances, before an intervention is imperative if remote delivery methods are to be used hereafter and reduce feelings of anxiety. A related facilitator was participants being familiar with the technology, thus reinforcing the need to ensure individuals have the correct level of digital skills required to engage. This facilitator has been highlighted in previous research on remote engagement with interventions as being a crucial component to not only engagement but also intervention satisfaction, and subsequently adherence [88–90]. Increasing skills through education and training would also align with the finding that those with a higher education had greater engagement, and those with a lower education had greater disengagement. This is supported by a range of previous research which highlights a positive association between education level and digital literacy in studies that have examined remote health interventions [91, 92]. Previous research has displayed that lower digital literacy skills have also been positively associated with older age, which is another barrier this review highlighted to engaging in remote weight management interventions [93, 94].

The results of this review also suggest that remote engagement enables individuals with functional or mobility issues to access services which they would usually be unable to. This corresponds with the results of a multitude of other studies examining remote interventions in health [95–98]. It is noteworthy that individuals with audio or visual impairments exhibited limited physical capability to engagement. This particularly becomes a barrier for those with visual impairments when there may be certain visual cues from peers or professionals that are not being received via remote intervention. Previous research that was conducted during COVID-19 among individuals with visual impairments suggested that some visual cues were missed and more detailed descriptions were required for any infographics used, thus making communication more difficult for both provider and participant [99]. Opposingly, other research has indicated that some video platforms can support individuals with visual impairments in real-time through methods such as screen readers [100, 101]. Henceforth, the mode of delivery should be carefully considered by intervention providers to ensure appropriate support for individuals with physical capability limitations.

The social opportunity provided, allowing connectedness with peers was the most frequently stated facilitator to engagement. It was also observed that a lack of social support and peer-connectedness was a barrier to engagement. This aligns with other research in the areas of health interventions that indicates increased social support has a positive effect on engagement and adherence [90, 102]. Not only this, but research also suggests that social support within health interventions improves the outcomes targeted by the intervention [103, 104].

The results of this review highlight the importance of recognising health inequalities across ethnic minority groups. Relative to Caucasians, research has evidenced the health inequalities of individuals from ethnic minority groups, with black adults in particular being at greater risk of obesity [105]. Many studies demonstrate the disproportionate effects of overweight and obesity among ethnic minority groups, even though there is evident underrepresentation of ethnic minority groups within weight management studies [106, 107]. It is noteworthy in this study that 30% of the included papers did not report ethnicity; and of those that did report ethnicity, only 25% were identified as having an ethnically diverse sample (≥50% from an ethnic minority group). An inadequate representation of individuals from ethnic minority groups has been demonstrated in previous reviews, with only 33% from ethnic minority groups (18% African American, 9% Hispanic/Latino, 5% Asian and 1% Native American) and 8% categorised as “Other” [108]. Thus, future research should ensure to not only report the ethnicity of the sample but to include a more ethnically diverse and representative sample of the research population. Regarding effectiveness, a related systematic review [109] has demonstrated that eHealth approaches display feasible short-term efficacy with modest weight loss in comparison to control groups for those from ethnic minority groups, albeit that this was from a small study collation (*N* = 6). Accordingly, even greater emphasis should be placed on the inclusion of an ethnically diverse sample in future research.

A common barrier to remote interventions related to both motivation and the physical opportunity to engage, linked to digital exclusion. The mitigation of this has been previously proposed through the use of assessing digital literacy and providing face-to-face demonstrations before the intervention, increasing confidence to use technology platforms, utilising user-friendly platforms, and increasing access to good internet connection, software and technology [42, 85].

### Strengths and limitations

This is the first known study to systematically review the barriers and facilitators that influence adults engaging in a remote weight management intervention, drawing on a COM-B behavioural analysis. This review consists of a large sample size with several methodological approaches, therefore increasing the generalisability of the study results. This study accounted for the adjusted BMI for ethnic minorities by including studies with a BMI ≥ 27.5 kg/m^2^. It included interventions provided in many countries, using numerous delivery techniques and to a wide-ranging sample. However, the findings from this review should be interpreted with the following limitations in mind. Studies that reported demographic predictors which in some cases could be considered either a barrier or facilitator to engagement were excluded. Minimal grey literature was found and, it was beyond the scope of this review to determine relationships between influences and intervention effectiveness, therefore changes in BMI have not been reported. Another limitation, due to the focus of the review being barriers and facilitators to uptake and engagement, this study did not extract the impacts of engagement and retention on weight loss, or the potential implications from anti-obesity medications.

## Conclusion

This review has provided a novel contribution to the literature to help understand factors that influence adults’ engagement with remote weight management interventions, addressing a gap in the literature [110]. It has provided a synthesis of studies in this area, the majority of which were deemed high quality, and furthered the interpretation of findings by mapping thematic influences to the COM-B model [38] of behaviour change. This, in turn, offers insight that can be used by intervention designers to optimise engagement in future weight management interventions. Alongside this, it is recommended that when developing, delivering or evaluating future weight management interventions, the barriers presented from the COM-B analysis in this review should be considered and mitigated where possible to lack of engagement and health inequalities. Furthermore, it is important to optimise ‘what works’, therefore, future interventions should also utilise as many of the facilitators identified in this paper as possible to provide an evidence-based foundation for remote weight management interventions. As weight loss itself was not a focus of this review, and therefore not reported in some of the papers included due to their study design (e.g. qualitative), future research should consider the effects of intervention retention on weight loss. Future research should also investigate the effects of anti-obesity medications on engagement and retention to such interventions.
